# Physical activity, sedentary leisure-time and risk of incident type 2 diabetes: a prospective study of 512 000 Chinese adults

**DOI:** 10.1136/bmjdrc-2019-000835

**Published:** 2019-12-18

**Authors:** Derrick A Bennett, Huaidong Du, Fiona Bragg, Yu Guo, Neil Wright, Ling Yang, Zheng Bian, Yiping Chen, Canqing YU, Sisi Wang, Fanwen Meng, Jun Lv, Junshi Chen, Liming Li, Robert Clarke, Zhengming Chen, on behalf of the China Kadoorie Biobank Study Collaborative Group, Junshi Chen, Zhengming Chen, Robert Clarke, Rory Collins, Yu Guo, Liming Li, Jun Lv, Richard Peto, Robin Walters, Daniel Avery, Derrick Bennett, Ruth Boxall, Fiona Bragg, Yumei Chang, Yiping Chen, Zhengming Chen, Robert Clarke, Huaidong Du, Simon Gilbert, Alex Hacker, Michael Holmes, Christiana Kartsonaki, Rene Kerosi, Garry Lancaster, Kuang Lin, John McDonnell, Iona Millwood, Qunhua Nie, Pang Yao, Paul Ryder, Sam Sansome, Dan Schmidt, Rajani Sohoni, Iain Turnbull, Robin Walters, Lin Wang, Neil Wright, Ling Yang, Xiaoming Yang, Zheng Bian, Yu Guo, Xiao Han, Can Hou, Biao Jing, Chao Liu, Jun Lv, Pei Pei, Yunlong Tan, Canqing Yu, enzhu Tang, Naying Chen, Ying Huang

**Affiliations:** 1 Clinical Trial Service Unit and Epidemiological Studies Unit (CTSU), Nuffield Department of Population Health, University of Oxford, Oxford, UK; 2 Medical Research Council Population Health Research Unit (MRC PHRU), Nuffield Department of Population Health, University of Oxford, Oxford, UK; 3 Chinese Academy of Medical Sciences, Beijing, China; 4 Department of Epidemiology and Biostatistics, School of Public Health, Peking University Health Science Center, Beijing, China; 5 Non-Communicable Disease Prevention and Control Department, Liuzhou CDC, Liuzhou, Guangxi, China; 6 China National Center for Food Safety Risk Assessment (CFSA), Beijing, China

**Keywords:** physical activity, sedentary leisure-time, adiposity, type 2 diabetes

## Abstract

**Objective:**

Aim to examine the independent and joint associations of physical activity (PA) and sedentary leisure-time (SLT) with risk of diabetes and assess the extent to which these associations were mediated by adiposity.

**Research design and methods:**

The prospective China Kadoorie Biobank recruited ~512 000 adults from 10 diverse areas across China. Self-reported PA was estimated based on type, frequency and duration of specific types of PA, covering four domains (occupation, leisure, household and commuting). SLT was defined as hours per day spent watching television, reading or playing card games. Stratified Cox proportional hazards models were used to estimate adjusted HRs (aHRs) for PA and SLT associated with incident diabetes. Analyses were stratified by age-at-risk (5-year intervals), sex and region and adjusted for household income, education, alcohol consumption, smoking, fresh fruit intake, self-reported general health status, family history of diabetes and body mass index (BMI) status. Analyses of total PA, occupational and non-occupational PA and SLT were mutually adjusted for each other, as appropriate.

**Results:**

After ~9 years of follow-up, there were 14 940 incident diabetes cases among 460 736 participants without prior diabetes or cardiovascular diseases at baseline. The mean (SD) age at baseline was 51 (10.6) years, 59% were women and 43% resided in urban areas. Overall, the mean BMI was 23.5 (3.3) kg/m^2^, which differed by ~0.5 kg/m^2^ among individuals in the highest compared with the lowest PA and SLT groups. PA was inversely associated the risk of diabetes 16% (aHR: 0.84, 95% CI 0.81 to 0.88) lower in top than bottom fifth. After further adjustment for BMI this was attenuated to 0.99 (95% CI 0.98 to 1.00). SLT was positively associated with diabetes and each 1 hour per day higher usual level was associated with aHR of 1.13 (95% CI 1.09 to 1.17) for diabetes, attenuated to 1.05 (95% CI 1.01 to 1.09) after further adjustment for BMI.

**Conclusions:**

Among Chinese adults, higher levels of PA and lower levels of SLT were associated with lower risks of diabetes with no evidence of effect modification by each other. These associations appeared to arise mainly through adiposity.

**Significance of this study**

**What is already known about this subject?**

- There is good evidence from observational studies in high-income countries that physical activity is inversely associated with risk of diabetes and that sedentary behavior is positively associated with diabetes risk.
- However, evidence is lacking from low-income and middle-income countries, and few studies have investigated both the independent and joint associations of physical activity and sedentary behavior with future risk of diabetes.

**What are the new findings?**

- Using data from the prospective China Kadoorie Biobank study, we assessed the associations of physical activity and sedentary leisure-time with incident diabetes cases among 460 736 participants without prior diabetes or cardiovascular diseases at baseline, among whom 14 940 developed diabetes.
- Higher levels of physical activity were associated with lower risks of diabetes, whereas the converse was true for higher levels of sedentary leisure-time.
- These associations of physical activity and sedentary leisure-time with risks of diabetes were independent of each other, but were more modest after additional adjustment for body mass index.

**How might these results change the focus of research or clinical practice?**

- These findings show that physical activity and sedentary leisure time may be important risk factors for diabetes, but these associations appear to be chiefly explained by adiposity.
- These results highlight the importance of research on lifestyle and behavioural changes to tackle the rising burden of obesity in different populations, which could impact on the burden of diabetes.

## Introduction

The prevalence of diabetes has increased substantially in recent decades, particularly in low-income and middle-income countries (LMICs), including China where about 10% of adults have diabetes.^1 2^ Observational studies conducted in high-income countries (HICs) have demonstrated that higher levels of physical activity (PA), typically measured through self-report, are associated with lower risks of diabetes.^3^ In contrast, higher levels of sedentary behavior are associated with higher risks of diabetes.^4^ Randomized trials in HICs have demonstrated that lifestyle modification, including both weight loss and increase in PA, can reduce both the short-term and long-term risks of type 2 diabetes (T2D).^5–8^

Previous studies of PA and diabetes have mainly assessed the disease associations with leisure-time PA, without taking appropriate account of other domains of PA including occupational, commuting and household PA. Rapid urbanization, reduction in PA in the work place and changes in modes of transport and other aspects of lifestyle have resulted in lower overall levels of PA globally,^9^ and changes in the patterns of PA both in China and in other LIMCs.^10^ Moreover, the pattern and overall levels of PA in China differ importantly from those in HICs, but the evidence about the independent relevance of PA and sedentary leisure-time (SLT) with risk of diabetes is limited.^11 12^

Several previous studies in China have examined the associations of PA with diabetes, but they were constrained by an insufficient number of cases,^13^ analyses restricted to single sex^14 15^ or restriction of analyses to specific domains of PA.^16 17^ Moreover, little is known in the worldwide literature about the relevance of occupational and non-occupational (including leisure-time) PA for risks of diabetes in population subgroups (eg, age, sex) and the potential role of adiposity as a mediator of such associations.^18^ Previous reports from China on sedentary behavior and diabetes have used mostly cross-sectional designs,^19^ and hence, there is a need for further prospective studies in China addressing these questions to guide public health strategies for the prevention of diabetes.

The aims of the present study were: (i) to examine the independent associations of PA (total, occupational and non-occupational) and of SLT with risk of diabetes, both overall and in certain population subgroups; (ii) to examine the joint associations of PA and SLT with risk of diabetes and (iii) to assess the extent to which these associations are mediated by adiposity.

## Methods

### Study population

This study is reported as per the Strengthening the Reporting of Observational studies in Epidemiology statement. Details of the study design and methods of the China Kadoorie Biobank (CKB) have been previously reported.^20–22^ Briefly, participants were recruited from 10 (5 urban and 5 rural) diverse areas in China, chosen from China’s nationally representative Disease Surveillance Points (DSP) system^23^ to maximize geographic and socioeconomic diversity (online supplementary eFigure 1). A total of 1 801 200 registered residents aged 35–74 years in the study areas were identified through local residential records and invited to attend study clinics between June 2004 and July 2008. Overall, 512 713 individuals (including 10 168 who were just outside the prespecified age range) were enrolled in the present study. All participants provided written informed consent.

10.1136/bmjdrc-2019-000835.supp1 Supplementary data

### Data collection

Trained health workers administered a laptop-based questionnaire on demographic, socioeconomic and lifestyle (eg, PA, smoking, diet) factors, and medical history, and undertook anthropometric and physical measurements (including blood pressure and lung function).^21^ A 10 mL non-fasting blood sample was collected from participants and random plasma glucose (RPG) levels were measured immediately (SureStep Plus; LifeScan, Johnson and Johnson). Time since last meal was recorded on all participants. Individuals with a plasma glucose level ≥7.8 and <11.1 mmol/L were invited back the following day to have fasting plasma glucose (FPG) concentrations measured. Individuals with self-reported doctor diagnosed diabetes (n=16 162) or with screen-detected diabetes (no self-reported diabetes, but RPG level ≥7.0 mmol/L with time since last eating ≥8 hours, or ≥11.1 mmol/L with time since last eating <8 hours, or an FPG level ≥7.0 mmol/L on subsequent testing, n=14 137)^24^ were excluded from the present analyses. Periodic re-surveys were conducted on ~5% of randomly selected surviving participants.

#### PA and sedentary behavior measurement

Details of the methods used to assess PA have been previously reported.^25 26^ In brief, the CKB PA questionnaire was based on previous validated questionnaires from high-income^27^ and Chinese^28^ populations and included questions on the intensity and frequency of, and time spent on, occupational, commuting, household and leisure-time activities (see detailed questionnaire at www.ckbiobank.org). Metabolic equivalents of tasks (METs) of different types of activities were adopted from the 2011 compendium of physical activities.^29^ The MET of each activity was multiplied by the frequency and duration of PA to calculate PA in MET hours per day (MET-h/day) from each activity (online supplementary eTable 1). Occupational PA included all PA performed during paid employment; non-occupational PA included all PA performed during travel to and from work, household activity and leisure-time exercise. Total PA was the summation of occupational and non-occupational PA.^25^ Time spent (in hours per day) on sedentary activities during leisure-time such as watching television, reading or playing card games was defined as SLT.

#### Follow-up for morbidity and mortality

Vital status of participants was monitored periodically through DSP death registries,^23^ supplemented by annual active confirmation of survival through local street committees or village administrators. The causes of death were coded by trained DSP staff using 10th revision of the International Statistical Classification of Diseases and Related Health Problems (ICD-10),^30^ blinded to the baseline information. Data on non-fatal disease outcomes were obtained by linkage, using the participant’s unique personal identification number, with established registries for major chronic diseases and with National Health Insurance claim systems, which provided almost universal (~99%) coverage of all hospitalisations for participants in the study. Incident cases of T2D (ICD-10: E10-E14) were identified by record linkage to health insurance databases, disease surveillance systems and death registries. By 1 January 2017, 37 289 participants (7.3%) had died and 4098 (0.8%) were lost to follow-up.

### Statistical analyses

Individuals with prevalent diabetes (n=30 299), a self-reported history of coronary heart disease (n=15 472), stroke or transient ischaemic attack (n=8884), rheumatic heart disease (n=937), those who reported implausibly extreme, or conflicting, levels of PA (n=1888) were excluded, leaving 460 736 participants for the present analyses.

Baseline characteristics of individuals were classified into quintiles for total PA and five groups for SLT and incident rates of diabetes in each group were calculated after adjustment for age (5-year groups), sex and region using direct standardization. Stratified Cox proportional hazards models were used to estimate HRs for incident T2D associated with quintiles of PA and five groups for SLT to ensure roughly equal numbers of participants in each group. The Cox regression analyses were stratified by age-at-risk (5-year intervals), sex and region (10 groups) and adjusted for household income, education, alcohol consumption (6 groups for each), smoking (4 groups), fresh fruit intake (5 groups), self-reported general health status (4 groups), family history of diabetes (dichotomous) and body mass index (BMI) status (3 groups). Age-at-risk is a time-varying covariate whereby as an individual gets older during follow-up they may contribute to more than one ‘age-at-risk’ group. Analyses of total PA, occupational and non-occupational PA and SLT were mutually adjusted for each other, as appropriate. The HRs and 95% CIs for quintiles of PA and groups of SLT were computed using group-specific variances, such that the HR in each group, including the reference group, is associated with a group-specific 95% CI.^31^ The proportional hazards assumptions for the Cox model were assessed using standard methods.^32^

The associations of PA and SLT with diabetes were corrected for regression dilution bias using repeat PA and SLT data collected at re-survey among ~20 000 participants (conducted ~3 years after the baseline survey).^33^ The regression dilution ratios (RDRs) were calculated using the McMahon-Peto method that estimates the RDR.^33^ Log HRs per 4 MET-h/day (equivalent to 1 hour walking/day) higher baseline PA were then multiplied by the reciprocal of the RDRs to obtain HRs (and associated 95% CI) for 4 MET-h/day usual PA with diabetes. For SLT, log HRs per 1 hour/day higher baseline SLT were multiplied by the reciprocal of the RDR to obtain associations of 1 hour/day *usual* SLT with diabetes. To facilitate a direct comparison between PA and SLT findings, we also calculated the HRs per 1 SD higher usual PA and usual SLT, respectively.

The joint associations of total PA and SLT were assessed by creating nine groups based on tertiles of both total PA and SLT and using Cox regression to estimate the HRs of diabetes for each group. The joint association of PA/SLT and BMI were assessed by creating six groups based on tertiles of total PA or SLT and two BMI groups (<25, ≥25 kg/m^2^) due to limited numbers of participants with BMI >30 kg/m^2^. Cox regression analysis of PA and SLT were performed separately within BMI groups (<25, ≥25 kg/m^2^), gender and area (urban, rural) to assess potential effect modification by adiposity, gender and area. The proportion of diabetes risk associated with PA and SLT explained by BMI was estimated based on the difference between log HRs for models with and without adjustment for BMI.^34^

Sensitivity analyses were performed after excluding participants with type 1 diabetes, participants who reported other chronic diseases (eg, cancer, respiratory diseases), or poor self-rated health at baseline, and cases of diabetes diagnosed during the first 3 years of follow-up. In addition, the impact of sequential adjustment for several lifestyle factors, dietary and physical measurements on the association of total PA and SLT with risk of diabetes was also examined. All analyses used two sided p values and were conducted using SAS V.9.2 and R V.3.4.2.

## Results

Among the 460 736 participants, the mean (SD) age at baseline was 51 (10.6) years, the mean BMI was 23.5 kg/m^2^, 59% were women and 43% resided in urban areas (table 1). Individuals with higher levels of total PA had less SLT, were more likely to be male, younger, living in rural areas and engaged in more manual work. Such individuals also had lower levels of BMI, waist circumference, per cent body fat, blood pressure and heart rate, and had better self-rated health (table 1). For SLT, the associations with these baseline characteristic factors appeared to mirror those for PA (online supplementary eTable 2).

**Table 1:** Selected baseline characteristics by level of physical activity

Characteristic	Baseline total physical activity (MET-h/day)					All
	<9.40	9.40–14.99	15.00–22.81	22.82–33.99	34.00+	
Number of participants	91 095	91 482	93 788	92 190	92 181	460 736
Physical activity-related factors						
Total physical activity, MET-h/day	6.2 (2.5)	12.3 (1.6)	18.6 (2.3)	28.0 (3.2)	42.7 (8.1)	21.8 (13.8)
Sedentary leisure-time, hour/day	3.6 (1.8)	3.3 (1.5)	3.0 (1.4)	2.7 (1.4)	2.6 (1.3)	3.0 (1.5)
Demographic factors						
Age, years	57.8 (11.0)	54.0 (10.6)	50.2 (9.9)	47.8 (9.1)	46.1 (8.2)	51.2 (10.5)
Women, %	60.4	65.9	61.5	53.9	45.2	59.0
Living in urban area, %	50.7	44.5	43.9	34.4	29.9	42.3
Socioeconomic and lifestyle factors, %						
High school education or above	18.2	23.9	24.9	21.2	16.5	20.8
Household income ≥¥20 000 per year	37.0	45.1	45.8	43.6	40.4	42.7
Manual worker	27.1	44.4	62.4	71.2	74.3	58.8
Ever regular smoker: men	76.3	74.6	72.8	73.9	74.6	74.6
Women	3.7	3.0	2.8	2.8	2.8	3.0
Current drinker: men	32.1	35.4	34.7	34.8	34.4	34.0
Women	2.0	2.1	2.2	2.5	2.6	2.1
Daily fresh fruit consumption	18.6	20.5	20.4	18.2	16.5	18.2
Daily fresh vegetable consumption	95.2	96.0	94.8	93.0	95.1	94.7
Daily meat/poultry consumption	30.7	30.7	29.1	28.1	27.8	28.9
Daily fish consumption	2.8	3.1	3.1	2.7	2.4	2.7
Daily soybean product consumption	3.6	3.8	4.0	4.0	3.6	3.5
Physical and blood measurements						
BMI, kg/m^2^	23.7 (3.6)	23.7 (3.5)	23.5 (3.3)	23.3 (3.2)	23.3 (3.1)	23.5 (3.3)
Waist circumference, cm	80.7 (10.1)	80.4 (9.7)	79.8 (9.5)	79.2 (9.3)	78.9 (8.9)	79.7 (9.6)
Body fat percentage	28.5 (9.0)	28.3 (8.5)	27.7 (8.1)	27.2 (7.9)	27.0 (7.8)	27.7 (8.3)
Obese, %	4.5	4.0	3.5	2.9	2.7	3.5
SBP, mm Hg	130.1 (22.6)	130.0 (21.5)	129.7 (20.3)	129.6 (19.9)	129.4 (19.1)	130.0 (20.8)
Heart rate, bpm	79.6 (11.9)	79.1 (11.5)	78.5 (11.5)	78.2 (11.7)	77.6 (11.7)	78.6 (11.7)
Random plasma glucose, mmol/L	5.7 (1.2)	5.7 (1.2)	5.7 (1.1)	5.7 (1.1)	5.7 (1.1)	5.7 (1.1)
Self-reported conditions at baseline, %						
Hypertension*	10.4	10.2	8.8	8.1	7.3	9.1
Poor health	13.2	9.1	7.8	7.7	7.4	8.8
Family history of diabetes†	6.3	6.8	6.7	6.5	6.0	6.3

Values are mean (SD) unless otherwise stated. Means and percentages are directly standardised to age, sex and study area structure of the included study population, as appropriate.

Two participants with missing BMI data; 214 participants with missing body fat percentage data; 7884 participants with missing random plasma glucose data.

Obese, BMI ≥30 kg/m^2^.

*28.8% of participants had screen detected hypertension (SBP ≥140 mm Hg or DBP ≥90 mm Hg) at baseline.

†First-degree relatives only.

BMI, body mass index; DBP, diastolic blood pressure; MET-h/day, metabolic equivalents of task hours per day; SBP, systolic blood pressure.

### Physical activity and diabetes risk

**Figure 1:**
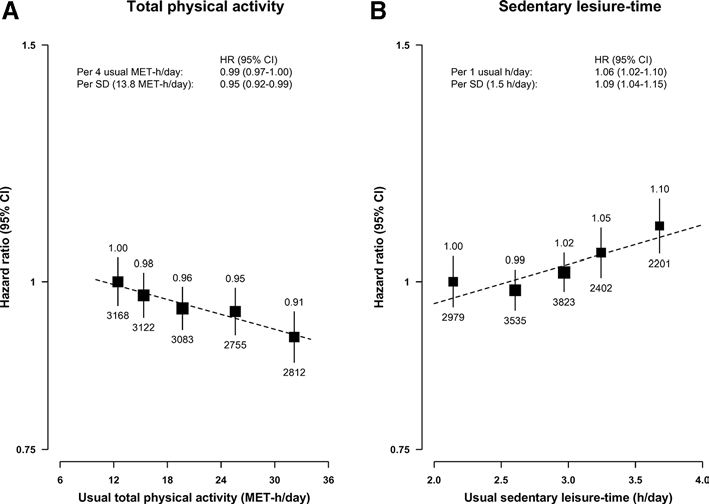
Adjusted HRs for risk of new-onset diabetes by total physical activity and by sedentary leisure-time. All analyses were stratified by age-at-risk, sex and region and adjusted for household income, education, smoking, alcohol, fresh fruit consumption, self-rated health, family history of diabetes, body mass index status (<25, 25–29, 30+ kg/m²) and sedentary leisure-time or total physical activity as appropriate. Results per 1 SD increase are for usual total physical activity and sedentary leisure-time. The size of the squares are proportional to the inverse variance of each effect size. The dashed line represents the slope from a weighted linear regression with weights based on the inverse variance of the log HRs. MET-h/day, metabolic equivalents of task hours per day.

During 4.3 million person-years (mean ~9 years) of follow-up, 14 940 (2.8%) incident cases of diabetes were recorded among those aged 35–79 years. Total PA was inversely associated with risk of diabetes, with adjusted HRs being 1.00, 0.96, 0.91, 0.89 and 0.84 from lowest to the highest fifth groups (p_trend_ <0.0001; model 2 in table 2), which were independent of SLT. Each 1 SD (13.8 MET-h/day) higher baseline total PA was associated with 5% lower risk of diabetes (HR=0.95, 95% CI 0.93 to 0.97), translating into 2% (HR=0.98, 95% CI 0.98 to 0.99) lower risk per 4 MET-h/day higher baseline total PA. By applying the RDRs (0.52 for total PA; online supplementary eTable 3), each 4 *usual* MET-h/day higher total PA was associated with 0.97 (95% CI 0.96 to 0.98) lower risk of diabetes (table 2) and this was attenuated to 0.99 (95% CI 0.97 to 1.00) after adjustment for BMI status (figure 1A, online supplementary eFigure 2a). These inverse associations, including effects of adjusting for BMI, were similar for occupational and non-occupational PA (HR per 4 usual MET-h/day: 0.98 (95% CI 0.96 to 0.99) vs 0.97 (95% CI 0.94 to 1.00), respectively before adjusting for BMI status and 0.99 (95% CI 0.97 to 1.00) vs 0.99 (95% CI 0.96 to 1.02), respectively after adjustment for BMI status; online supplementary eFigure 3). After further adjusting for BMI levels, there was a further small attenuation (model 4, table 2), suggesting that ~67% of the association of total PA with diabetes could be explained by differences in BMI.

**Table 2:** Adjusted rates and HRs for diabetes by baseline total physical activity and sedentary leisure-time

	No. of cases	Model 1		Model 2*		Model 3†		Model 4‡	
		Rate§	HR (95% CI) per 1000	Rate	HR (95% CI) per 1000	Rate	HR (95% CI) per 1000	Rate	HR (95% CI) per 1000
Baseline total physical activity (MET-h/day)									
<9.40	3168	3.73	1.00 (0.96 to 1.04)	3.61	1.00 (0.96 to 1.04)	3.47	1.00 (0.96 to 1.04)	3.42	1.00 (0.96 to 1.04)
9.40–14.99	3122	3.51	0.94 (0.91 to 0.98)	3.46	0.96 (0.92 to 0.99)	3.39	0.98 (0.94 to 1.01)	3.38	0.99 (0.95 to 1.02)
15.00–22.81	3083	3.27	0.88 (0.85 to 0.91)	3.29	0.91 (0.88 to 0.94)	3.32	0.96 (0.92 to 0.99)	3.33	0.97 (0.94 to 1.01)
22.82–33.99	2755	3.14	0.84 (0.81 to 0.88)	3.20	0.89 (0.85 to 0.92)	3.30	0.95 (0.91 to 0.99)	3.33	0.97 (0.93 to 1.01)
34.00+	2812	2.95	0.79 (0.76 to 0.82)	3.04	0.84 (0.81 to 0.88)	3.16	0.91 (0.87 to 0.95)	3.18	0.93 (0.89 to 0.97)
Trend	p<0.0001	p<0.0001		p=0.0010		p=0.0142
Per 4 MET-h/day increase	14 940	0.98 (0.97 to 0.98)	0.98 (0.98 to 0.99)	0.99 (0.99 to 1.00)	0.99 (0.99 to 1.00)
Per 1 SD (13.8 MET-h/day) increase	14 940	14 9400.93 (0.91 to 0.95)	0.95 (0.93 to 0.97)	0.97 (0.96 to 0.99)	0.98 (0.96 to 1.00)
Usual total physical activity									
Per 4 usual MET-h/day increase	14 940	0.96 (0.95 to 0.97)	0.97 (0.96 to 0.98)	0.99 (0.97 to 1.00)	0.99 (0.98 to 1.00)
Per 1 SD (13.8 MET-h/day) increase	14 940	0.87 (0.83 to 0.90)	0.90 (0.87 to 0.94)	0.95 (0.92 to 0.99)	0.96 (0.93 to 1.00)
Baseline sedentary leisure-time (hour/day)									
<1.5	2979	3.08	1.00 (0.96 to 1.04)	3.12	1.00 (0.96 to 1.04)	3.26	1.00 (0.96 to 1.04)	3.29	1.00 (0.96 to 1.04)
1.5–2.4	3535	3.11	1.01 (0.98 to 1.05)	3.14	1.01 (0.97 to 1.04)	3.21	0.99 (0.95 to 1.02)	3.23	0.98 (0.95 to 1.01)
2.5–3.4	3823	3.31	1.08 (1.04 to 1.11)	3.32	1.06 (1.03 to 1.10)	3.31	1.02 (0.98 to 1.05)	3.30	1.00 (0.97 to 1.03)
3.5–4.4	2402	3.51	1.14 (1.09 to 1.19)	3.50	1.12 (1.08 to 1.17)	3.42	1.05 (1.01 to 1.09)	3.40	1.03 (0.99 to 1.08)
4.5+	2201	3.89	1.26 (1.21 to 1.32)	3.78	1.21 (1.16 to 1.27)	3.58	1.10 (1.05 to 1.15)	3.54	1.08 (1.03 to 1.13)
Trend		P<0.0001	P<0.0001	P=0.0002	P=0.0032
Per 1 hour/day increase	14 940	1.05 (1.04 to 1.06)	1.04 (1.03 to 1.05)	1.02 (1.01 to 1.03)	1.02 (1.00 to 1.03)
Per 1 SD (1.5 hour/day) increase	14 940	1.07 (1.06 to 1.09)	1.06 (1.04 to 1.08)	1.03 (1.01 to 1.05)	1.02 (1.01 to 1.04)
Usual sedentary leisure-time									
Per 1 usual hour/day increase	14 940	1.16 (1.12 to 1.20)	1.13 (1.09 to 1.17)	1.06 (1.02 to 1.10)	1.05 (1.01 to 1.09)
Per 1 SD (1.5 hour/day) increase	14 940	1.25 (1.19 to 1.31)	1.20 (1.13 to 1.26)	1.09 (1.04 to 1.15)	1.07 (1.02 to 1.13)

All analyses were stratified by age-at-risk, region and sex.

*Model 2 was additionally, on top of model 1, adjusted for household income, education, smoking, alcohol, fresh fruit consumption, self-rated health, family history of diabetes and sedentary leisure-time or physical activity as appropriate.

†Model 3 was additionally, on top of model 2, adjusted for BMI status (<25, 25-29, 30+kg/m^2^).

‡Model 4 was additionaly, on top of model 3, adjusted for BMI.

§ Rates are estimated from model hazard ratios.

MET-h/day, metabolic equivalents of task hours per day.

### Sedentary leisure-time and diabetes risk

In contrast, SLT had a positive log-linear association with diabetes, with the adjusted HRs being 1.00, 1.01, 1.06, 1.12 and 1.20 across five groups (p_trend_ <0.0001; table 2). Each 1 hour higher baseline SLT was positively associated with a 4% (adjusted HR=1.04, 95% CI 1.03 to 1.05) higher risk, which was independent of total PA. After adjusting for regression dilution (RDR 0.32 for SLT) each 1 hour higher usual SLT was positively associated with 13% (HR=1.13, 95% CI 1.09 to 1.17) higher risk (figure 1B, online supplementary eFigure 2b). The HR was attenuated to 1.06 (95% CI 1.02 to 1.10) after adjustment for BMI status and to 1.05 (95% CI 1.01 to 1.09) after further adjustment for BMI levels, suggesting that ~60% of the association of SLT with diabetes could be explained by differences in BMI (table 2).

### Associations of PA and SLT with diabetes by gender and area

**Figure 2:**
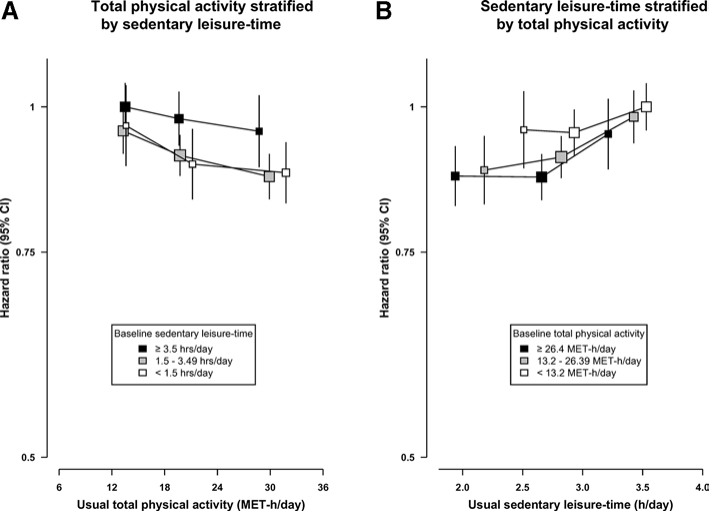
Joint associations of total physical activity and sedentary leisure-time with risk of diabetes. All analyses were stratified by age-at-risk, sex and region and adjusted for household income, education, smoking, alcohol, fresh fruit consumption, self-rated health, family history of diabetes and body mass index status (<25, 25–29, 30+ kg/m²). The size of the squares are proportional to the inverse variance of each effect size. MET-h/day, metabolic equivalents of task hours per day.

The strength of the inverse associations of total, occupational and non-occupational PA was broadly similar for men and women and residents living in urban and rural areas (online supplementary eFigures 4a–7a). For SLT, however, the strength of the positive association was about threefold greater in men (HR per 1 SD: 1.17 (95% CI 1.07 to 1.27)) as in women (HR per 1 SD: 1.05 (95% CI 0.98 to 1.13)) (online supplementary efigure 4b) and for participants living in rural (HR per 1 SD: 1.15 (95% CI 1.06 to 1.25)) than in urban (HR per 1 SD: 1.05 (95% CI 0.98 to 1.13)) areas (online supplementary eFigure 5b). The inverse association of usual total PA with diabetes was similar across different levels of SLT (figure 2A) and usual SLT was positively associated with diabetes risk at all levels of PA (figure 2B).

### Effect modification of PA and SLT associations with diabetes by BMI

When stratified by BMI, the inverse association of PA with diabetes was only statistically significant in those individuals with BMI ≥25 kg/m^2^ (HR per 4 usual MET-h/day: 0.98 (95% CI 0.97 to 0.99)), and not in those with BMI <25 kg/m^2^ (1.00 (95% CI 0.98 to 1.01); online supplementary eFigure 8a), although the difference was not statistically significant p_heterogeneity_=0.15. The approximately log-linear positive association for SLT with diabetes risk was similar in those individuals with BMI <25 kg/m^2^ and with BMI ≥25 kg/m^2^ (HR per 4 usual MET-h/day: 1.07 (95% CI 1.01 to 1.13) vs 1.06 (95% CI 1.01 to 1.11), respectively; online supplementary eFigure 8b).

### Combined effects of PA and SLT with BMI on risk of diabetes

When considering combined effects of PA and BMI on the risk of diabetes, the HR was 3.20 (95% CI 3.07 to 3.33) among individuals who were both overweight and had low PA compared with those with high PA and normal weight (online supplementary eFigure 9). In normal weight individuals, the low PA group had 13% higher risk than high PA group. Among individuals who were both overweight and had high SLT the HR was 3.11 (95% CI 2.99 to 3.23) compared with those with low SLT and normal weight. Normal weight individuals with high SLT group had 8% (95% CI 3% to 14%) higher risk than the normal weight individuals with low SLT (online supplementary eFigure 10)

### Associations of PA and SLT with diabetes by subgroups

The strength of the inverse association of total PA with diabetes was broadly similar in subgroups defined by education, income, levels of alcohol consumption, smoking status and systolic blood pressure (SBP (online supplementary eFigure 11). However, the effects of total PA appeared to be modified by family history of diabetes (p=0.01). For SLT there was some evidence of a trend by age-at-risk (p=0.04), but there was no evidence of differences in the strength of association by sex, levels of education, income, alcohol consumption, smoking status or SBP. There was, however, some evidence of heterogeneity by occupation (p<0.01, online supplementary eFigure 11).

There was no clear evidence of heterogeneity in the inverse association of total PA with risk of diabetes across the five urban regions (p=0.09), or five rural regions (p=0.19; online supplementary eFigure 12). For SLT, there was no evidence of heterogeneity within the urban regions (p=0.41), but borderline statistically significant heterogeneity between rural regions; p=0.04, respectively (online supplementary eFigure 12).

### Sensitivity analyses

Additional adjustment for dietary variables, or separate exclusion of cases of T2D diagnosed during the first 3 years of follow-up, participants with a self-reported history of chronic diseases at baseline, or participants with poor self-rated health or disability had little effect on the overall inverse associations with diabetes for total, occupational and non-occupational PA, and positive associations for SLT, respectively (online supplementary eTable 4).

The impact of progressive adjustment for individual confounders and measures of adiposity on the inverse associations of the top versus bottom groups of PA and positive association with SLT is presented in online supplementary eFigure 13. Additional adjustment for waist circumference on top of BMI levels yielded HRs of 0.96 (95% CI 0.90 to 1.01) and 1.06 (95% CI 0.99 to 1.13) for PA and SLT, respectively.

## Discussion

Using data from the CKB higher levels of total PA were inversely associated with risk of diabetes, while the converse was true for SLT. Each 1 SD higher usual PA and usual SLT was associated with 5% lower and 9% higher risk of diabetes, respectively, and the associations appeared largely independent of each other and broadly similar across different population subgroups. However, adjustment for BMI greatly attenuated the strength of the associations, suggesting that the observed associations of PA and SLT with risk of diabetes were due to a combination of confounding and mediation by adiposity. To our knowledge, this is one of the largest studies to investigate both the independent and joint effects of PA and SLT with risk of diabetes.

Several prospective studies of populations in HICs have examined the associations of PA and SLT with risk of diabetes.^4 35 36^ In general, they tended to show inverse and positive associations for PA and SLT, respectively, but the strength of the reported associations varied substantially, due perhaps chiefly to heterogeneity in the measurements of PA and SLT and variation sample sizes. The findings of the present study are less extreme than those reported from a recent meta-analysis of 14 prospective studies in HICs involving 104 908 participants and 18 276 T2D cases. This meta-analysis reported that individuals with high self-reported PA (estimated based on a combination of methods from the included studies) had a 35% lower risk of diabetes compared with those with low PA.^37^ Within our single study population, we were able to ensure consistency of measurement of PA, whereas many studies included in the meta-analysis used different definitions for PA (eg, MET-hours, steps per week or simply reported low, moderate and high categories), which prevented a reliable dose-response analysis,^37^ and direct comparison with findings from the present study.

The findings from the present study on SLT derived from a single population were broadly consistent with those of a previous meta-analysis of prospective studies that examined the effects of sedentary behaviour using a similar classification of SLT (television watching) to that used in our study. The meta-analysis included 400 292 participants and 17 552 incident cases of T2D and reported that one extra hour/day television watching measured at baseline was associated with 9% (95% CI 7% to 12%) higher risk of diabetes after adjustment for PA.^38^ Few previous studies have simultaneously examined the associations of PA and SLT with risk of diabetes in the same population. In a small multiethnic population study of 5829 people (non-Hispanic whites, Chinese-Americans, African-Americans and Hispanic Americans) with 655 incident cases of diabetes, sedentary behavior had a stronger association with diabetes risk than total PA but the associations for both PA and SLT varied by ethnicity with weaker effects in the Chinese and other non-white groups.^39^

Several previous studies have also assessed the mediating effects of adiposity and showed reasonably consistently that much of the association of PA and SLT with risk of diabetes was explained by adiposity, defined almost exclusively by BMI.^4 37 40 41^ A meta-analysis of 10 cohort studies from mainly HICs where the mean BMI was high reported a pooled HR of 0.69 for moderate intensity PA without adjustment for BMI, which was attenuated to 0.83 after adjustment for BMI, suggesting about 50% of the association was explained by adiposity.^42^ This appeared somewhat smaller than that shown in the present study (~50% vs 67%) involving a population with much lower mean BMI and a wider range of PA. Previous evidence for adiposity explaining the association between SLT and diabetes risk is conflicting. For example, the US Black Women’s Health Study with 2928 diabetes cases found that the association of television watching with diabetes was attenuated by ~20% after adjustment for BMI,^40^ while the US Health Professional’s Follow-up Study, which included 1058 new cases of diabetes, found that prolonged television watching remained strongly positively associated with diabetes risk after adjustment for BMI,^43^ contrary to the present study findings.

Few large prospective studies have presented age-specific and sex-specific results for the association of PA and SLT with diabetes mutually adjusted for each other, with and without adjustment for BMI. The present study showed that while the associations of PA with diabetes appeared similar across different age groups and in men and women, the associations of SLT with diabetes were more extreme at younger than older ages and in men than in women. Such differences have not been thoroughly explored previously and require further replication in other populations.

The overall mean levels of total PA in our study were higher than those seen in HICs (due to the higher levels of occupational PA), but were similar to other East Asian populations such as those in a recent Japanese cohort of middle-aged adults.^44^ However, the overall mean levels of SLT (~3.0 hours/day) in the CKB were lower than those seen in an English population (mean age 65; mean television viewing time ~5 hours/day).^41^ Apart from the potential mediating effects via adiposity, PA may have short-term and long-term independent favorable effects on diabetes through other mechanisms.^45 46^ For example, acute PA may increase insulin-stimulated glucose uptake into active skeletal muscle,^47^ which accounts for 80% of insulin-stimulated glucose disposal. Likewise, long-term PA may improve insulin action, glycemic control or fat oxidation and storage in skeletal muscle.^47^ Sedentary behavior such as television watching could also have some impact on diabetes risk by reducing energy expenditure resulting in unfavorable effects on energy balance.^36^ There is good evidence of an association between television watching and higher energy intake.

The present study has several strengths, including large sample size, reliable assessment of risks associated with several domains of PA, occupational and non-occupational PA and SLT, both overall and in relevant population subgroups. The analyses excluded individuals with previously diagnosed and screen-detected diabetes at baseline, to reduce the potential for reverse causality. The quality of diagnosis for new-onset T2D was high. A medical record review for about 1000 incident cases of T2D confirmed the validity of the diagnosis (positive predictive value 97%, based on American Diabetes Association diagnostic criteria).^48^ The analyses conducted adjusted for a wide range of potential confounding factors in addition to correction for regression dilution bias.

However, the study also had several limitations. First, although validity of diabetes diagnoses was high, incident diabetes was restricted to hospital diagnosed cases (diabetic medication use information was not available), which may have resulted in some underdiagnosis. Second, reliance on measurement of random plasma glucose (supplemented by fasting time data) at baseline to identify and exclude those prevalent cases of diabetes (rather than fasting or oral glucose tolerance testing), might fail to identify some prevalent cases of diabetes. However, the prevalence of diabetes at baseline in CKB was comparable with that in a contemporaneous nationally representative survey in China.^49^ Third, as in all previously reported studies, we only collected self-reported data on PA and SLT, which would be subject to reporting bias, especially compared with objectively quantified PA and sedentary behavior, which have now been increasingly applied in epidemiological studies in recent years. Fourth, we did not collect detailed information on sedentary time at work, television-related and non-television-related SLT, or cardiorespiratory fitness, which could help to further reduce residual confounding. However, in the current study exclusion of participants with poor self-reported health at baseline did not alter the results. Fifth, although the risk estimates were corrected for regression dilution bias, we were unable to correct for measurement errors in covariates. It is therefore possible that the observed associations may still be affected by residual confounding due to suboptimally measured factors, as well as unknown, or unmeasured factors. Finally, whether adiposity is a mediator or confounder for PA and SLT cannot be fully determined in this study.

The implications of the present study findings are that PA and SLT are modestly associated with T2D, but this could be important at a population level. Participants should be encouraged to endeavor to engage in more PA of any type (either occupational or non-occupational) and to engage in less sedentary activities such as television watching in order to lower their risk of T2D.^50^ Further studies in China and other LMICs are needed to determine whether adiposity is a mediator or confounder for these associations, as well as understanding other mechanisms by which PA and SLT could relate to diabetes risk.

In conclusion, among Chinese adults, higher levels of PA and SLT had opposing and independent associations with risks of diabetes. While the beneficial effects of PA on diabetes appeared similar across different population subgroups, the adverse effects of SLT were more extreme in younger than older participants and in men than in women. The associations of PA and SLT with risk of diabetes could be chiefly mediated by adiposity, highlighting the importance of lifestyle and behavioral changes to tackle the rising burden of obesity in different populations.
