# Supplementary material for: Physical activity, sedentary leisure-time and risk of incident type 2 diabetes: a prospective study of 512 000 Chinese adults
Source: BMJ Open Diabetes Res Care. 2019 Dec 18;7(1):e000835. doi: 10.1136/bmjdrc-2019-000835 (PMC6936425; doi:10.1136/bmjdrc-2019-000835)
Supplement: Supplementary data [file bmjdrc-2019-000835supp001.pdf]

Supplementary Material For Physical activity, sedentary leisure-time, and risk of incident diabetes: a prospective study of 512,000 Chinese Adults

Derrick A. Bennett PhD[1]*, Huaidong Du PhD[1,2]*, Fiona Bragg DPhil[1]*, Yu Guo MSc[3], Neil Wright PhD[1], Ling Yang PhD[1,2], Zheng Bian MSc[3], Yiping Chen DPhil[1,2], Canqing Yu PhD[5], Sisi Wang BSc[6], Fanwen Meng BSc[6], Junshi Chen MD[4], Liming Li MPH[3,5], Robert Clarke FRCP[1], Zhengming Chen DPhil[1,2], on behalf of the China Kadoorie Biobank Study Collaborative Group (members listed at end of report)

*Contributed equally

[1] Clinical Trial Service Unit and Epidemiological Studies Unit (CTSU), Nuffield Department of Population Health, University of Oxford, Oxford, UK

[2] Medical Research Council Population Health Research Unit (MRC PHRU), Nuffield Department of Population Health, University of Oxford, Oxford, UK

[3] Chinese Academy of Medical Sciences, Beijing, China

[4] China National Center For Food Safety Risk Assessment, Beijing, China

[5] Department of Epidemiology and Biostatistics, School of Public Health, Peking University Health Science Center, Beijing, China

[6] Non-Communicable Disease Prevention and Control Department, Liuzhou CDC, NO.1-1 Tanzhong West Road, Liunan District, Liuzhou, Guangxi, China

Bennett DA, *et al. BMJ Open Diab Res Care* 2019;0:e000835. doi: 10.1136/bmjdrc-2019-000835

Page

3 eTable 1: Physical activity types, MET values, codes and intensity categories

4 eTable 2: Selected baseline characteristics by levels of sedentary leisure-time

5 eTable 3: Estimated regression dilution ratios for total, occupational and non-occupational physical activity and sedentary leisure-time overall and by measures of adiposity

6 eTable 4: Sensitivity analyses: Adjusted HRs for diabetes by total, occupational and non-occupational physical activity and sedentary leisure-time (i) additionally adjusted for dietary variables, (ii) excluding the first three years of follow-up, (iii) excluding those with chronic disease at baseline and (iv) excluding those with self-reported poor health

7 eFigure 1: Locations of the Kadoorie China Biobank recruitment centres

8 eFigure 2: Adjusted hazard ratios (HRs) for risk of new onset diabetes by total physical activity and sedentary leisure-time

9 eFigure 3: Adjusted HRs for diabetes by occupational and non-occupational physical activity

10 eFigure 4: Adjusted HRs for diabetes by total physical activity and sedentary leisure-time by sex

11 eFigure 5: Adjusted HRs for diabetes by total physical activity and sedentary leisure-time by area

12 eFigure 6: Adjusted HRs for diabetes by occupational and non-occupational physical activity by sex

13 eFigure 7: Adjusted HRs for diabetes by occupational and non-occupational physical activity by area

14 eFigure 8: Adjusted HRs for diabetes by total physical activity and sedentary leisure-time by BMI

15 eFigure 9: Joint associations of total physical activity and BMI with risk of diabetes

16 eFigure 10: Joint associations of sedentary leisure-time and BMI with risk of diabetes

17 eFigure 11: Adjusted HRs for diabetes per 4 MET-h/day increase in usual physical activity and per 1 h/day increase in usual sedentary leisure-time by subgroup

18 eFigure 12: Adjusted HRs for diabetes per 4 MET-h/day increase in usual physical activity and per 1 h/day increase in usual sedentary leisure-time by region

19 eFigure 13: Adjusted HRs for diabetes with total physical activity and sedentary leisure-time after progressive adjustments

eTable 1: Physical activity types, MET values, codes and intensity categories*

Activity type	Intensity	MET	Codes*
Heavy manual work	Vigorous	6.5	11477
Manual work	Moderate	4.5	11476
Standing work	Moderate	3.8	Mean of 11610 and 11630
Sedentary work	Low	1.8	Mean of 11580, 11585, and 11590
Manual work in the farming season	Vigorous	6.3	Mean of 11145 and 11146
Semi-mechanized work in the farming season	Moderate	3.4	Mean of 11146 and 11147
Fully mechanized work in the farming season	Low	2.4	Mean of 11147 and 11170
Work outside the farming season	Low	2	11147
Walking	Moderate	4	17270
Bicycle	Vigorous	6.8	1011
Motorbike	Moderate	3.5	16030
Private or public transportation (such as bus, car, underground, and ferry)	Low	1.7	Mean of 16010, 16015, and 16016
Household activity	Low	2.8	Mean of 05030[†], 05040[†], 05035, 05055, 05070, 05090[†], 05092[†], 05184, 05197, and 05200
Tai-Chi/qigong/leisure walking	Moderate	3.3	Mean of 15670 and 17160
Jogging/aerobic exercise	Vigorous	7.4	Mean of 03015, 12020, and 12150
Ball games	Moderate	5.5	Mean of 15020[†], 15030[†], 15055, 15080, 15090, 15255, 15605[†], 15610[†], 15652, 15660, 15675, 15710[†], and 15711[†]
Brisk walking/gymnastics/folk dancing	Moderate	4.2	Mean of 03025, 15300, and 17200
Swimming	Vigorous	7.2	Mean of 18230, 18240, and 18310
Other exercise, e.g. mountain walking, home exercise and rope jumping	Moderate	5.9	Mean of 02010, 02064, 04001, 04100, 15110[†], 15120[†], 15200, 15240, 15310, 15425[†], 15430[†], 15537, 15550[‡], 15551[‡], 15552[‡], 15580, 15590, 15730, 15732[‡], 15733[‡], 15734[‡], and 19030

MET: Metabolic equivalent of tasks.
* Based on the 2011 Compendium of Physical Activities: a second update of codes and MET values. Ainsworth BE, et al. Medicine and Science in Sports and Exercise, 2011;43(8):1575-1581.
[†] Assigned 1/2 weight in calculating the mean MET value because the connecting two items represent one type of activity.
[‡] Assigned 1/3 weight in calculating the mean MET value because the connecting three items represent one type of activity.

eTable 2: Selected baseline characteristics by levels of sedentary leisure-time

	Sedentary leisure-time (h/day)					
**Characteristic**	**≤1.5**	**1.5-2.5**	**2.5-3.5**	**3.5-4.5**	**≥4.5**	**All**
Number of participants	71,082	109,638	131,200	82,759	66,057	460,736
**Physical activity related factors**						
Total physical activity, MET-h/day	24.6 (15.3)	23.5 (14.1)	22.0 (13.4)	19.9 (12.7)	16.5 (11.5)	21.8 (13.8)
Sedentary leisure-time, h/day	1.0 (0.4)	2.0 (0.1)	3.0 (0.1)	4.0 (0.2)	5.6 (1.1)	3.0 (1.5)
**Demographic factors**						
Age, years	51.7 (10.6)	51.1 (10.3)	50.5 (10.3)	50.7 (10.5)	51.5 (11.0)	51.2 (10.5)
Female, %	63.6	60.1	55.5	55.7	55.8	59.0
Living in urban area, %	41.8	42.2	41.0	42.6	46.4	42.3
**Socio-economic and lifestyle factors, %**						
High school education or above	18.1	20.0	21.6	22.5	22.3	20.8
Household income ≥20,000 yuan/year	38.5	42.1	44.3	43.5	43.1	42.7
Manual worker	62.8	61.1	59.0	54.2	44.3	58.8
Ever regular smoker: Male	71.6	71.7	73.8	77.1	80.3	74.6
Female	2.7	2.7	2.9	3.1	4.4	3.0
Current drinker: Male	34.5	33.0	33.0	34.8	35.4	34.0
Female	2.4	2.1	2.0	2.3	2.7	2.1
Daily fresh fruit consumption	16.5	17.6	18.8	19.6	22.3	18.2
Daily fresh vegetable consumption	88.2	92.8	95.3	97.0	96.7	94.7
Daily meat/poultry consumption	27.2	28.4	29.2	30.9	33.6	28.9
Daily fish consumption	2.6	2.6	2.5	3.0	4.1	2.7
Daily soybean product consumption	3.7	3.6	3.3	3.4	3.9	3.5
**Physical and blood measurements**						
BMI, kg/m^2	23.3 (3.3)	23.4 (3.3)	23.6 (3.3)	23.7 (3.3)	23.9 (3.4)	23.5 (3.3)
Waist circumference, cm	78.8 (9.5)	79.3 (9.4)	79.9 (9.5)	80.3 (9.6)	80.9 (9.7)	79.7 (9.6)
Body fat percentage	27.0 (8.2)	27.4 (8.2)	27.8 (8.3)	28.2 (8.4)	28.7 (8.5)	27.7 (8.3)
Obese, %	2.9	3.1	3.5	3.9	4.7	3.5
SBP, mmHg	130.2 (22.0)	130.1 (20.8)	130.0 (20.4)	129.7 (20.4)	129.7 (20.7)	130.0 (20.8)
Heart rate, bpm	78.4 (11.8)	78.4 (11.6)	78.7 (11.6)	78.7 (11.6)	79.2 (11.7)	78.6 (11.7)
Random plasma glucose, mmol/L	5.7 (1.1)	5.7 (1.1)	5.7 (1.2)	5.6 (1.2)	5.6 (1.2)	5.7 (1.1)
**Self-reported conditions at baseline, %**						
Hypertension *	8.5	8.8	9.4	9.6	10.0	9.1
Poor health	10.3	9.1	8.0	8.1	10.5	8.8
Family history of diabetes †	5.8	6.0	6.5	6.9	7.4	6.3

Values are mean (SD) unless otherwise stated. Means and percentages are directly standardised to age, sex and study area structure of the included study population, as appropriate.
MET-h/day = Metabolic equivalents of task hours per day
BMI = Body mass index
SBP = Systolic blood pressure
Obese = BMI ≥ 30 kg/m2
* 28.8% of participants had screen detected hypertension (SBP ≥ 140 mmHg or diastolic blood pressure ≥ 90 mmHg) at baseline.
† 1st degree relatives only
2 participants with missing BMI data. 214 participants with missing body fat percentage data. 7,884 participants with missing random plasma glucose data.

eTable 3: Calculation of regression dilution ratios for total, occupational and non-occupational physical activity and sedentary leisure-time

	Baseline physical activity groups (MET-h/day)	Mean baseline physical activity (MET-h/day)	Mean first re-survey physical activity (MET-h/day)	Ratio of the ranges (MacMahon-Peto) regression dilution ratio estimate
**Total physical activity**				0.52
	≤9.39	6.09	12.46	
	9.40 - 14.99	12.18	15.34	
	15.00 - 22.81	18.56	19.68	
	22.82 - 33.99	28.22	25.59	
	≥34.00	43.68	32.18	
**Occupational physical activity**				0.50
	≤7.19	3.47	10.74	
	7.20 – 12.33	9.83	13.55	
	12.34 – 19.28	15.59	19.13	
	19.29 – 30.39	24.15	22.42	
	≥30.40	39.38	28.62	
**Non-occupational physical activity**				0.49
	≤3.92	2.12	4.57	
	3.93 – 6.49	5.29	6.72	
	6.50 – 8.39	7.37	7.65	
	8.40 – 11.52	9.67	9.18	
	≥11.53	15.51	11.17	

	Baseline sedentary leisure time (h/day)	Mean sedentary leisure time (h/day)	Mean first re-survey sedentary leisure time (h/day)	Ratio of the ranges (MacMahon-Peto) regression dilution ratio estimate
**Sedentary leisure time**				0.32
	<1.5	0.89	2.14	
	1.5-2.4	2.01	2.60	
	2.5-3.4	2.98	2.97	
	3.5-4.4	4.00	3.24	
	≥4.5	5.64	3.68	

Bennett DA, *et al. BMJ Open Diab Res Care* 2019;0:e000835. doi: 10.1136/bmjdrc-2019-000835

eTable 4: Sensitivity analyses: Adjusted HRs for diabetes by total, occupational and non-occupational physical activity and sedentary leisure-time (i) additionally adjusted for dietary variables, (ii) excluding the first three years of follow-up, (iii) excluding those with chronic disease at baseline and (iv) excluding those with self-reported poor health

	Adjusted for dietary variables *			Exclude first 3 years of follow-up			Exclude those with chronic disease at baseline †			Exclude those with self-reported poor health ‡		
	No. of events	Rate per 1000	HR (95% CI)	No. of events	Rate per 1000	HR (95% CI)	No. of events	Rate per 1000	HR (95% CI)	No. of events	Rate per 1000	HR (95% CI)
**Total physical activity (MET-h/day)**												
< 9.40	3,168	3.47	1.00 (0.96 - 1.04)	2,679	4.18	1.00 (0.96 - 1.04)	2,737	3.34	1.00 (0.96 - 1.04)	2,731	3.39	1.00 (0.96 - 1.04)
9.40 - 14.99	3,122	3.39	0.98 (0.94 - 1.01)	2,619	3.99	0.95 (0.92 - 0.99)	2,799	3.30	0.99 (0.95 - 1.03)	2,749	3.29	0.97 (0.93 - 1.01)
15.00 - 22.81	3,083	3.32	0.96 (0.92 - 0.99)	2,619	3.99	0.95 (0.92 - 0.99)	2,769	3.22	0.96 (0.93 - 1.00)	2,721	3.22	0.95 (0.92 - 0.99)
22.82 - 33.99	2,755	3.30	0.95 (0.92 - 0.99)	2,283	3.93	0.94 (0.90 - 0.98)	2,521	3.23	0.97 (0.93 - 1.01)	2,439	3.19	0.94 (0.90 - 0.98)
34.00 +	2,812	3.16	0.91 (0.87 - 0.95)	2,205	3.72	0.89 (0.85 - 0.93)	2,634	3.09	0.93 (0.89 - 0.97)	2,598	3.06	0.90 (0.86 - 0.94)
Total:	14,940			12,405			13,460			13,238		
Trend:			p=0.0012			p=0.0009			p=0.0120			p=0.0008
**Occupational physical activity (MET-h/day)**												
< 7.20	1,955	3.06	1.00 (0.95 - 1.06)	1,659	3.58	1.00 (0.94 - 1.06)	1,717	2.93	1.00 (0.94 - 1.06)	1,619	2.97	1.00 (0.94 - 1.06)
7.20 - 12.33	2,078	3.20	1.04 (1.00 - 1.09)	1,790	3.78	1.06 (1.01 - 1.11)	1,876	3.12	1.07 (1.02 - 1.12)	1,831	3.12	1.05 (1.00 - 1.10)
12.34 - 19.28	2,085	3.11	1.01 (0.97 - 1.06)	1,731	3.69	1.03 (0.98 - 1.08)	1,888	3.01	1.03 (0.98 - 1.07)	1,836	3.01	1.01 (0.97 - 1.06)
19.29 - 30.39	1,922	2.97	0.97 (0.93 - 1.01)	1,573	3.49	0.97 (0.93 - 1.02)	1,791	2.93	1.00 (0.96 - 1.05)	1,749	2.88	0.97 (0.92 - 1.02)
30.40 +	2,235	2.96	0.97 (0.92 - 1.01)	1,737	3.46	0.97 (0.92 - 1.02)	2,097	2.89	0.99 (0.94 - 1.04)	2,083	2.85	0.96 (0.91 - 1.01)
Total:	10,275			8,490			9,369			9,118		
Trend:			p=0.0374			p=0.0549			p=0.1579			p=0.0272
**Non-occupational physical activity (MET-h/day)**												
< 3.93	2,853	3.44	1.00 (0.96 - 1.04)	2,355	4.13	1.00 (0.95 - 1.05)	2,590	3.36	1.00 (0.96 - 1.05)	2,567	3.37	1.00 (0.96 - 1.05)
3.93 - 6.49	3,058	3.40	0.99 (0.95 - 1.02)	2,506	4.07	0.98 (0.95 - 1.02)	2,765	3.31	0.99 (0.95 - 1.02)	2,756	3.31	0.98 (0.95 - 1.02)
6.50 - 8.39	2,015	3.21	0.93 (0.89 - 0.97)	1,647	3.80	0.92 (0.88 - 0.97)	1,830	3.13	0.93 (0.89 - 0.98)	1,819	3.14	0.93 (0.89 - 0.98)
8.40 - 11.52	4,121	3.36	0.98 (0.94 - 1.01)	3,505	4.02	0.97 (0.94 - 1.01)	3,672	3.22	0.96 (0.93 - 0.99)	3,556	3.21	0.95 (0.92 - 0.99)
11.53 +	2,893	3.20	0.93 (0.89 - 0.97)	2,392	3.76	0.91 (0.87 - 0.95)	2,603	3.13	0.93 (0.89 - 0.97)	2,540	3.10	0.92 (0.88 - 0.96)
Total:	14,940			12,405			13.460			13,238		
Trend:			p=0.0215			p=0.0109			p=0.0169			p=0.0045
**Sedentary leisure-time (h/day)**												
< 1.5	2,979	3.26	1.00 (0.96 - 1.04)	2,203	3.80	1.00 (0.95 - 1.05)	2,722	3.18	1.00 (0.96 - 1.05)	2,691	3.17	1.00 (0.96 - 1.05)
1.5 - 2.4	3,535	3.21	0.98 (0.95 - 1.02)	2,925	3.84	1.01 (0.97 - 1.05)	3,223	3.13	0.98 (0.95 - 1.02)	3,174	3.12	0.98 (0.95 - 1.02)
2.5 - 3.4	3,823	3.31	1.02 (0.98 - 1.05)	3,272	3.94	1.04 (1.00 - 1.07)	3,432	3.18	1.00 (0.97 - 1.03)	3,432	3.22	1.01 (0.98 - 1.05)
3.5 - 4.4	2,402	3.41	1.05 (1.00 - 1.09)	2,099	4.11	1.08 (1.03 - 1.13)	2,163	3.34	1.05 (1.00 - 1.09)	2,121	3.33	1.05 (1.00 - 1.10)
4.5 +	2,201	3.58	1.10 (1.05 - 1.15)	1,906	4.28	1.12 (1.07 - 1.18)	1,920	3.48	1.09 (1.04 - 1.15)	1,820	3.43	1.08 (1.03 - 1.13)
Total:	14,940			12,405			13,460			13,238		
Trend:			p=0.0002			p<0.0001			p=0.0008			p=0.0021

All models stratified by age-at-risk, region and sex, and adjusted for household income, education, smoking, alcohol, fresh fruit consumption, self-rated health, family history of diabetes, BMI status (<25, 25-29, 30+ kg/m^2) and sedentary leisure-time.
* Also adjusted for frequency of meat consumption, frequency of dairy consumption and frequency of soybean product consumption.
† Exclude those with a self-reported history of TB, emphysema, bronchitis, asthma, cancer, kidney disease or rheumatoid arthritis at baseline.
‡ Exclude those with poor self-rated health or self-reported disability at baseline.

eFigure 1: Locations of the Kadoorie China Biobank recruitment centres

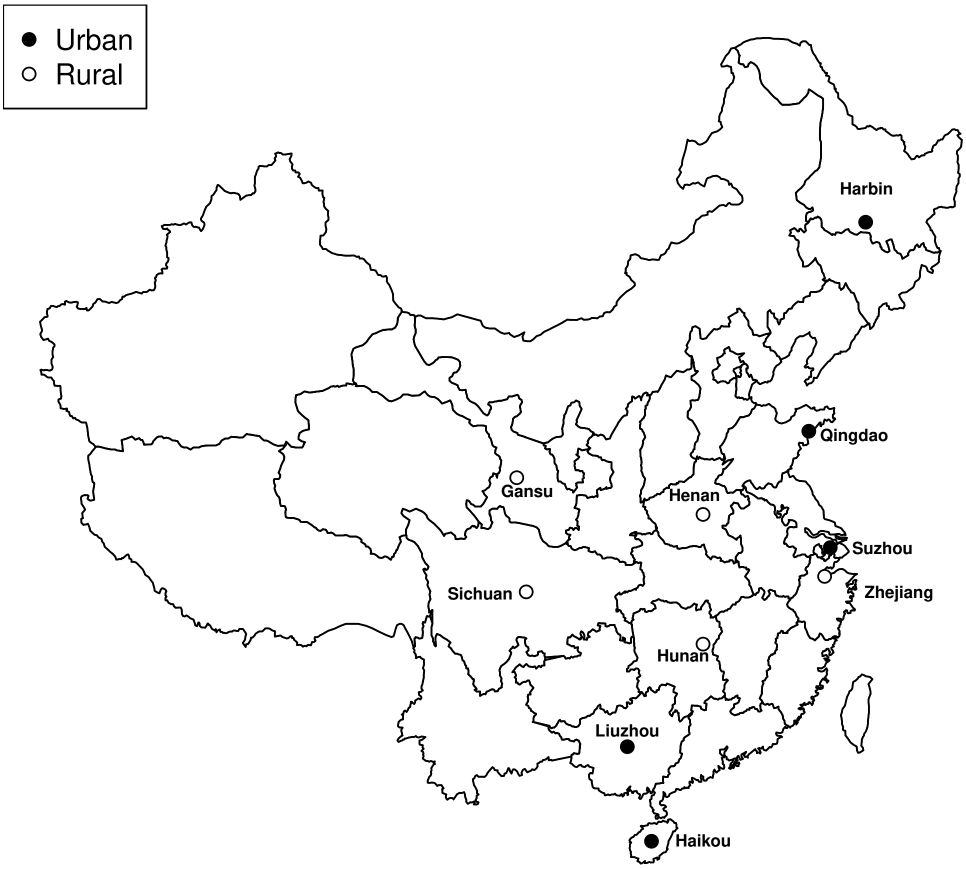

eFigure 2: Adjusted hazards ratios (HRs) for risk of new onset diabetes by total physical activity and sedentary leisure-time

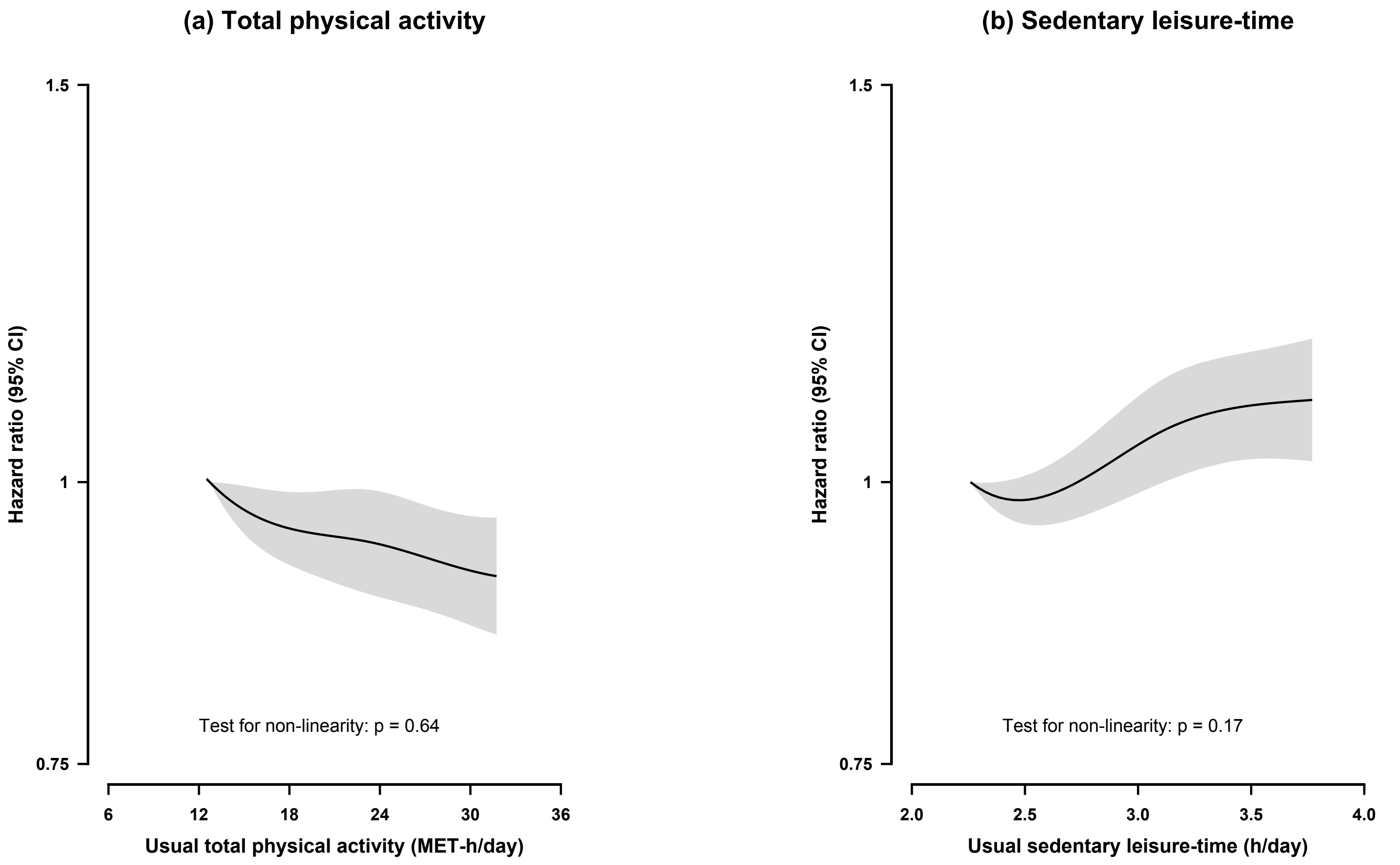

Bennett DA, *et al. BMJ Open Diab Res Care* 2019;0:e000835. doi: 10.1136/bmjdrc-2019-000835

eFigure 3: Adjusted HRs for diabetes by occupational and non-occupational physical activity

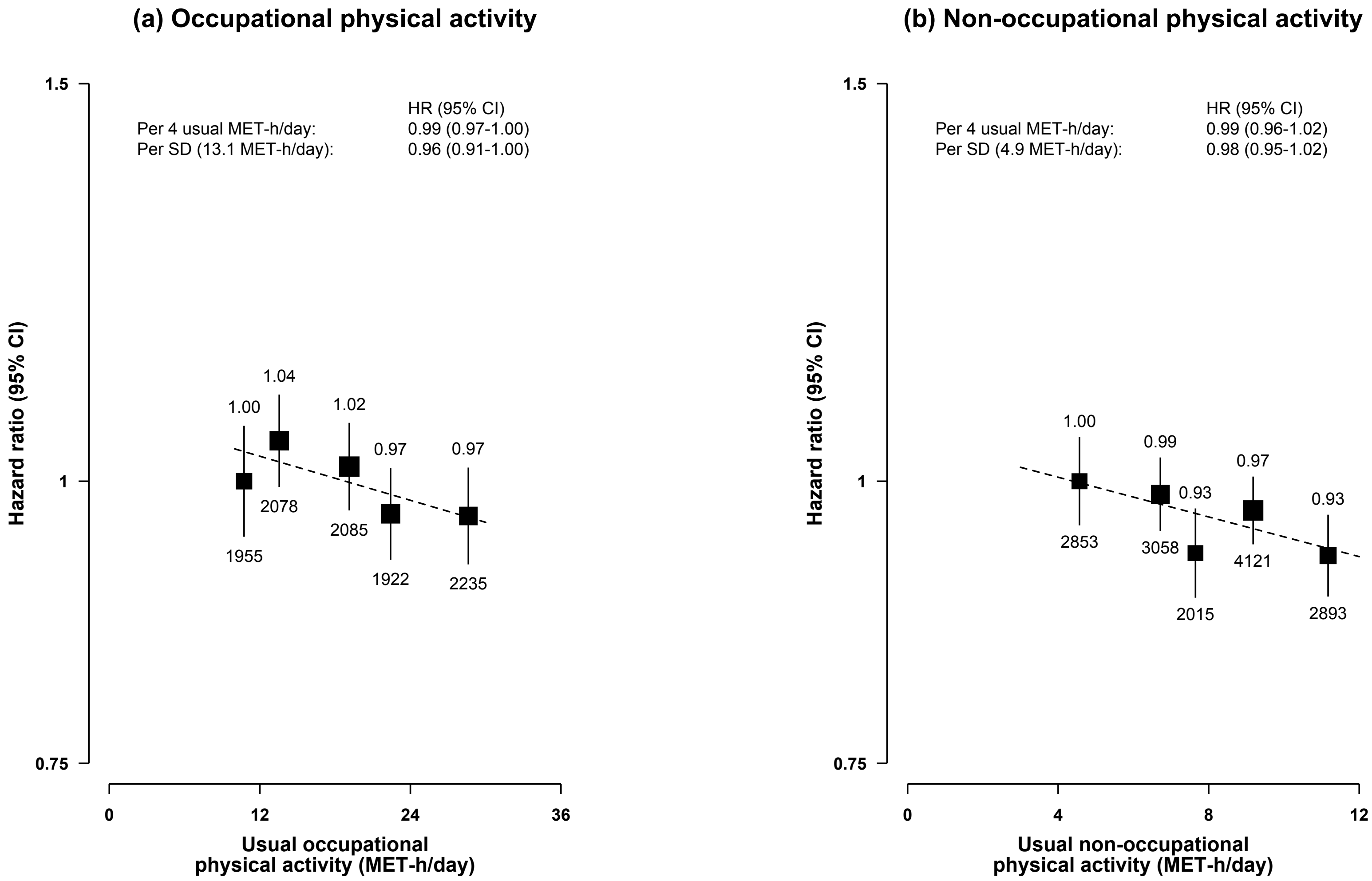

All analyses were stratified by age-at-risk, sex and region and adjusted for household income, education, smoking, alcohol, fresh fruit consumption, self-rated health, family history of diabetes, BMI status (<25, 25-29, 30+ kg/m²), sedentary leisure-time and occupational physical activity or non-occupational physical activity as appropriate.
Results per 1 SD increase are for usual occupational and non-occupational physical activity.

eFigure 4: Adjusted HRs for diabetes by total physical activity and sedentary leisure-time by sex

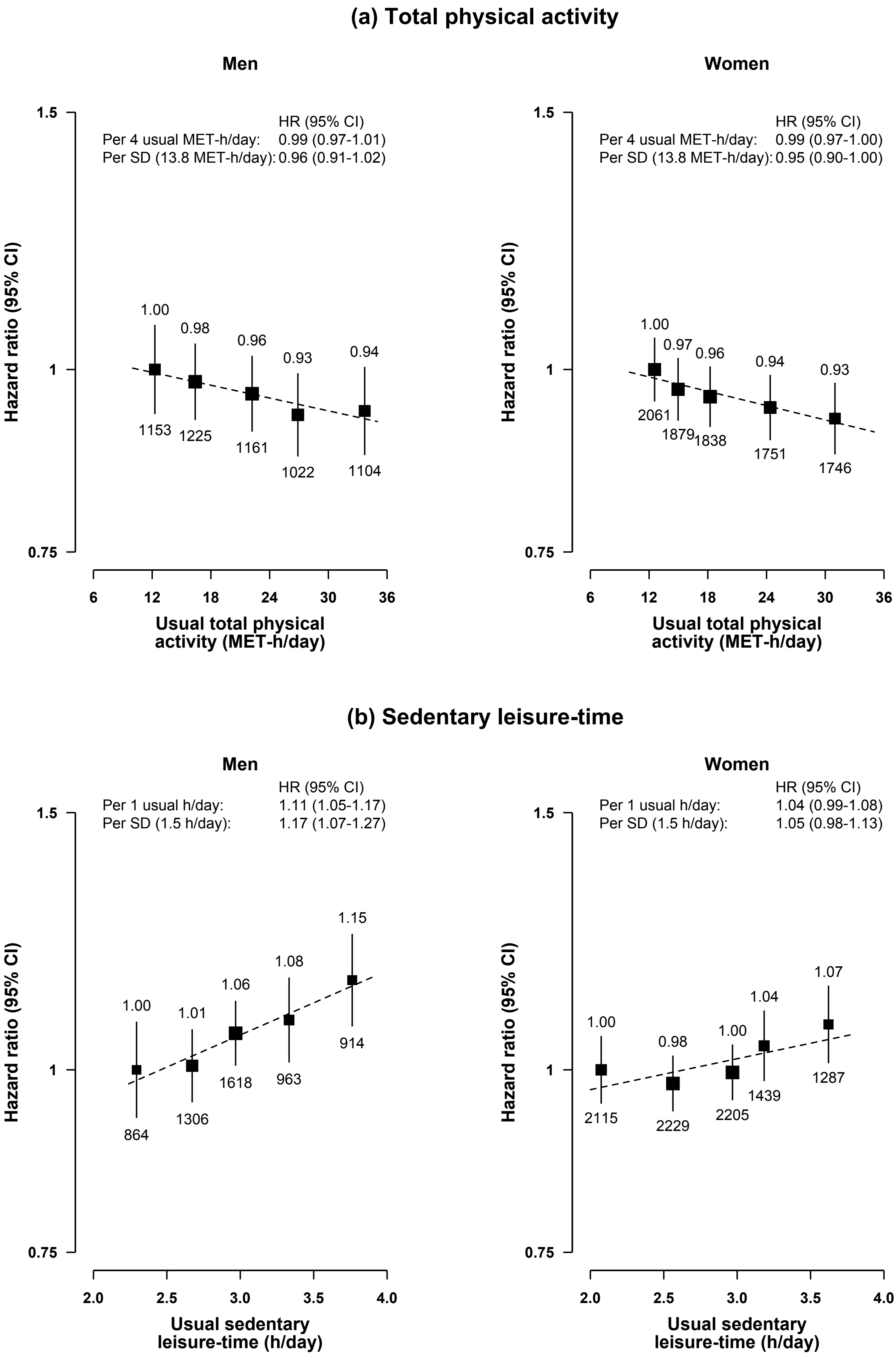

All analyses were stratified by age-at-risk and region and adjusted for household income, education, smoking, alcohol, fresh fruit consumption, self-rated health, family history of diabetes, BMI status (<25, 25-29, 30+ kg/m²) and sedentary leisure-time or total physical activity as appropriate. Results per 1 SD increase are for usual total physical activity and sedentary leisure-time.

eFigure 5: Adjusted HRs for diabetes by total physical activity and sedentary leisure-time by area

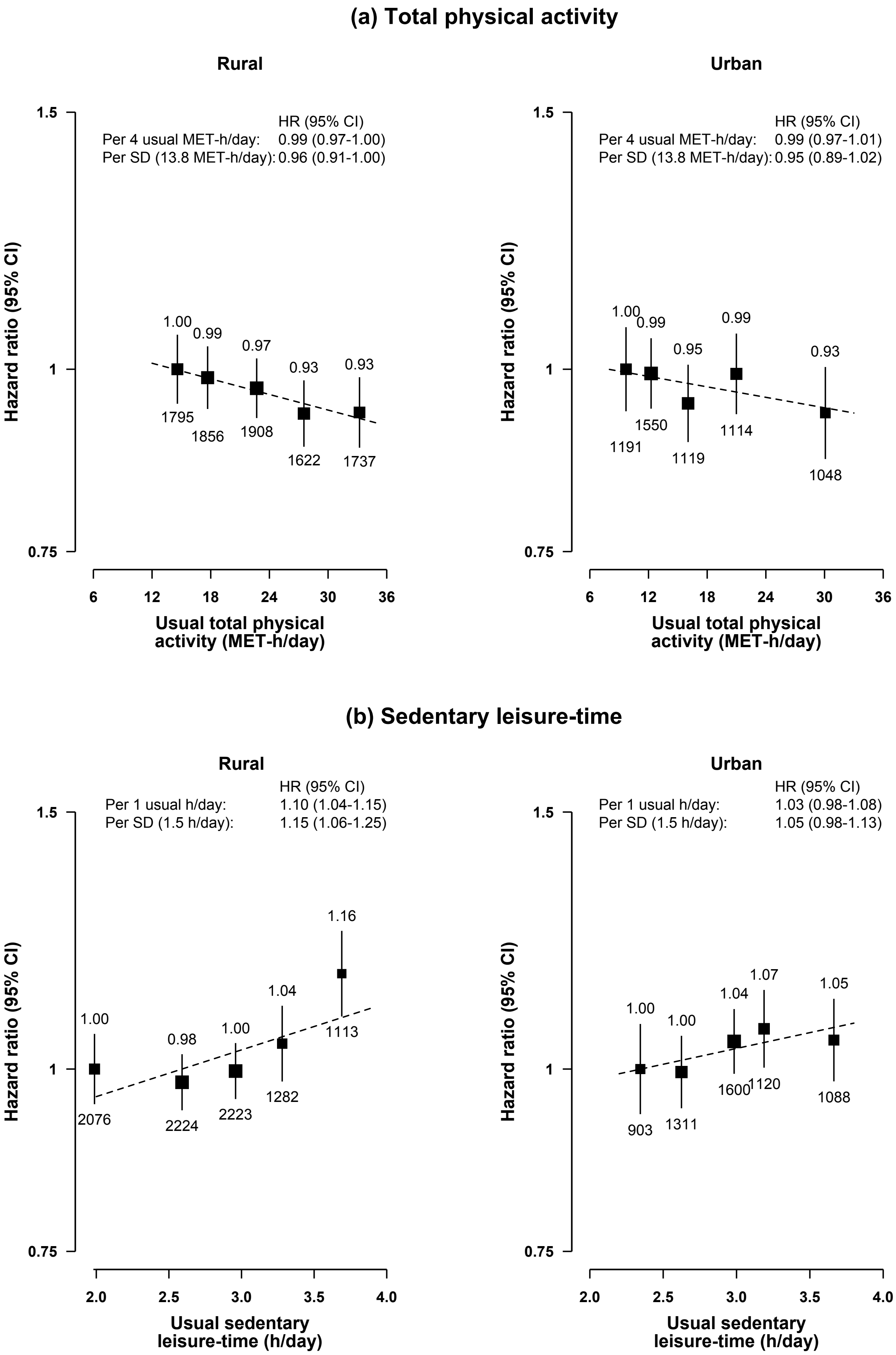

All analyses were stratified by age-at-risk, sex and region and adjusted for household income, education, smoking, alcohol, fresh fruit consumption, self-rated health, family history of diabetes, BMI status (<25, 25-29, 30+ kg/m²) and sedentary leisure-time or total physical activity as appropriate. Results per 1 SD increase are for usual total physical activity and sedentary leisure-time.

eFigure 6: Adjusted HRs for diabetes by occupational and non-occupational physical activity by sex

(a) Occupational physical activity

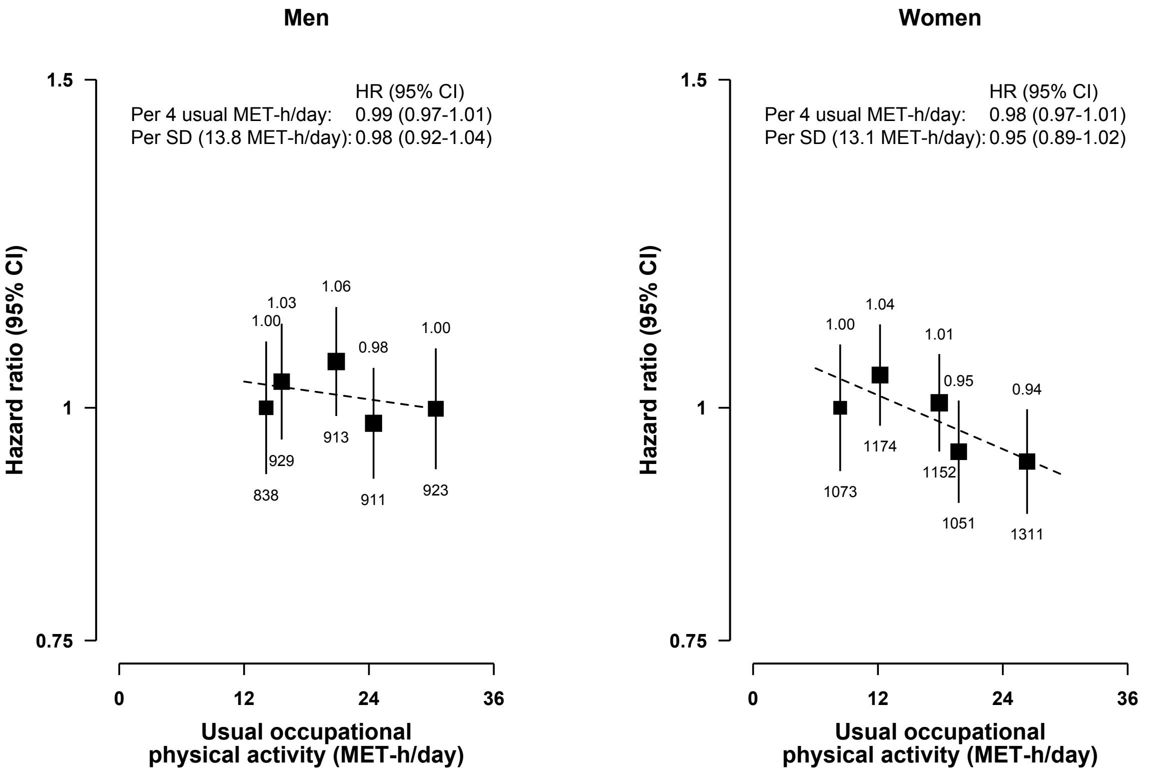

(b) Non-occupational physical activity

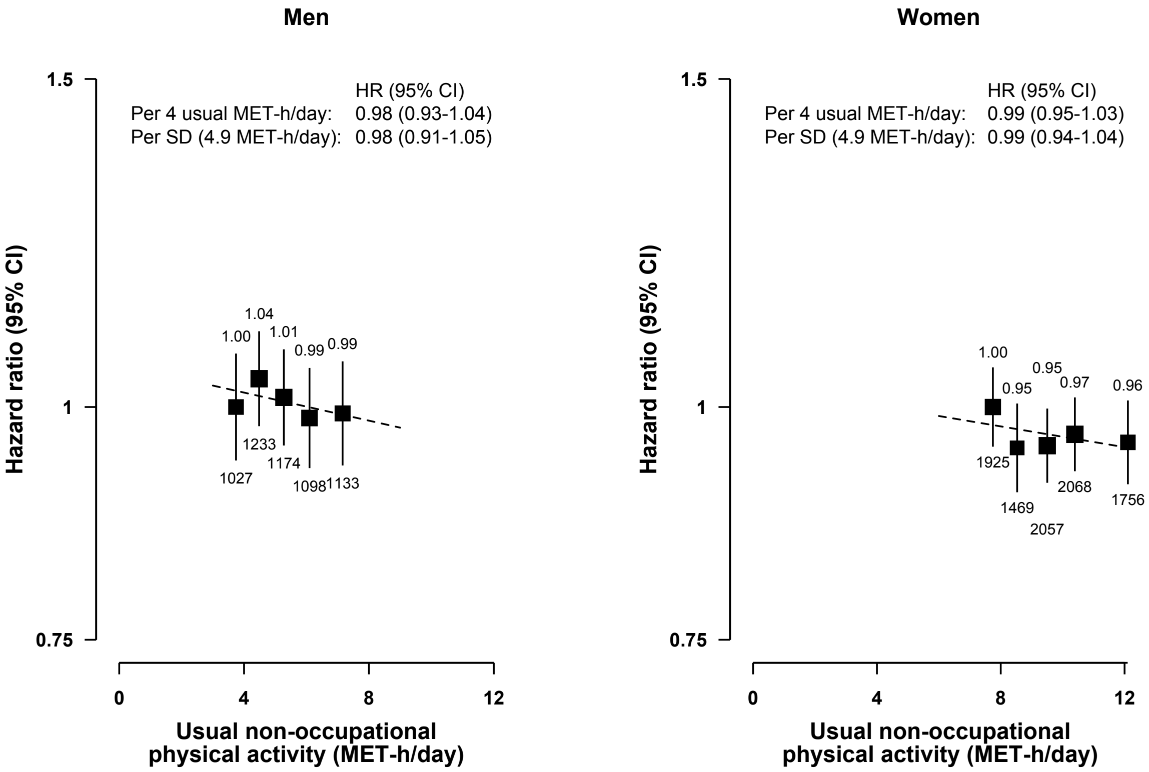

All analyses were stratified by age-at-risk and region and adjusted for household income, education, smoking, alcohol, fresh fruit consumption, self-rated health, family history of diabetes, BMI status (<25, 25-29, 30+ kg/m²) and non-occupational or occupational physical activity as appropriate. Results per 1 SD increase are for usual occupational and non-occupational physical activity.

eFigure 7: Adjusted HRs for diabetes by occupational and non-occupational physical activity by area

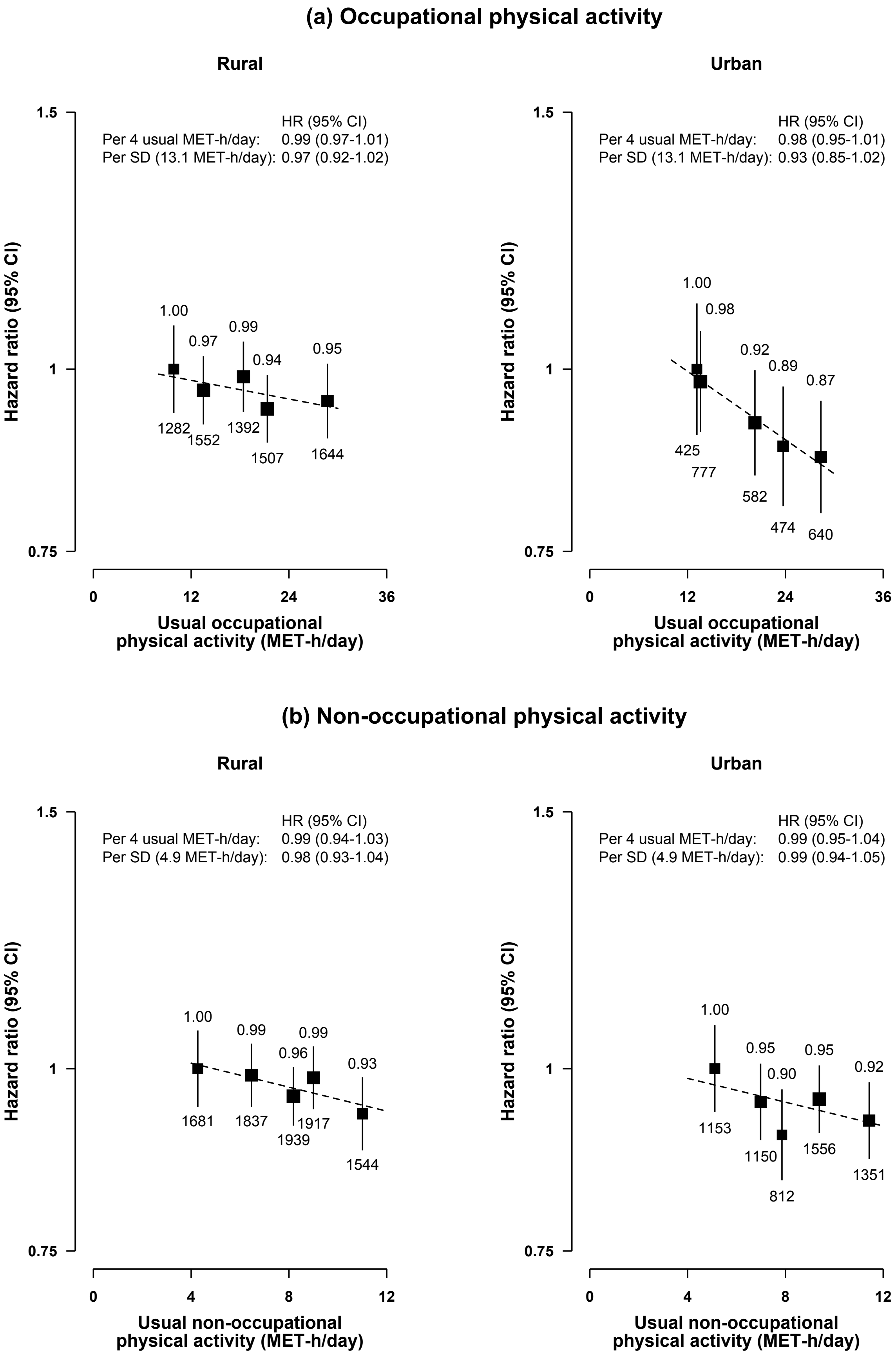

All analyses were stratified by age-at-risk, sex and region and adjusted for household income, education, smoking, alcohol, fresh fruit consumption, self-rated health, family history of diabetes, BMI status (<25, 25-29, 30+ kg/m²) and non-occupational or occupational physical activity as appropriate. Results per 1 SD increase are for usual occupational and non-occupational physical activity.

eFigure 8: Adjusted HRs for diabetes by total physical activity and sedentary leisure-time by BMI

(a) Total physical activity

BMI < 25 kg/m² **BMI ≥ 25 kg/m²**

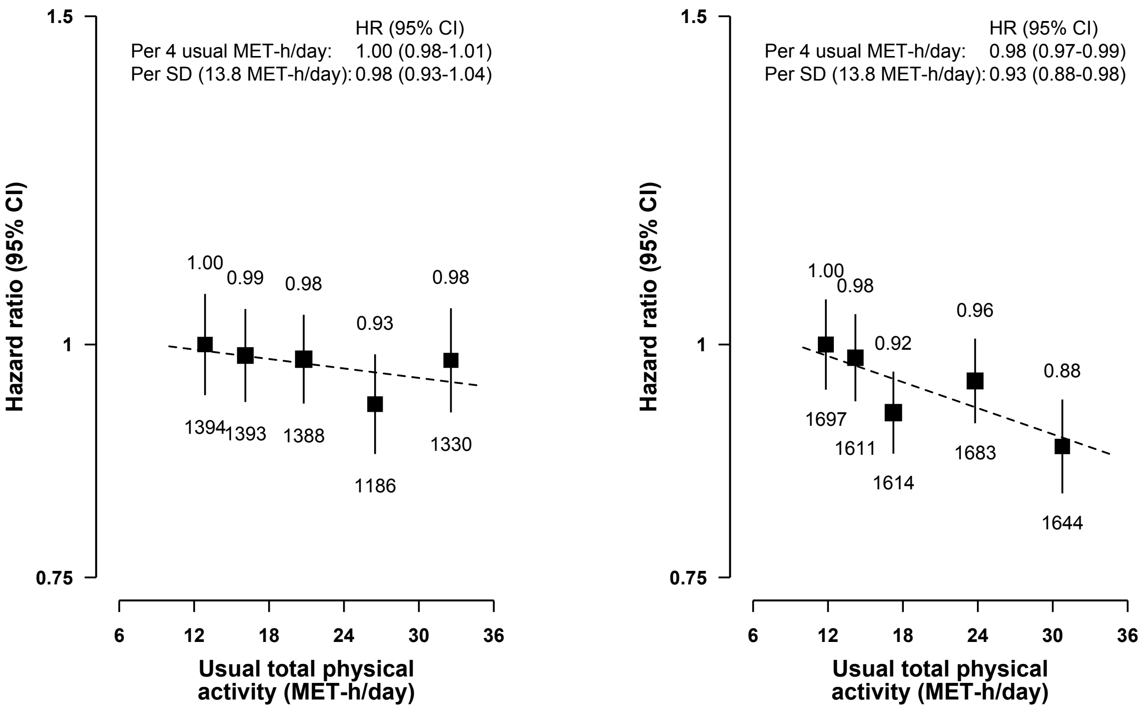

(b) Sedentary leisure-time

BMI < 25 kg/m² **BMI ≥ 25 kg/m²**

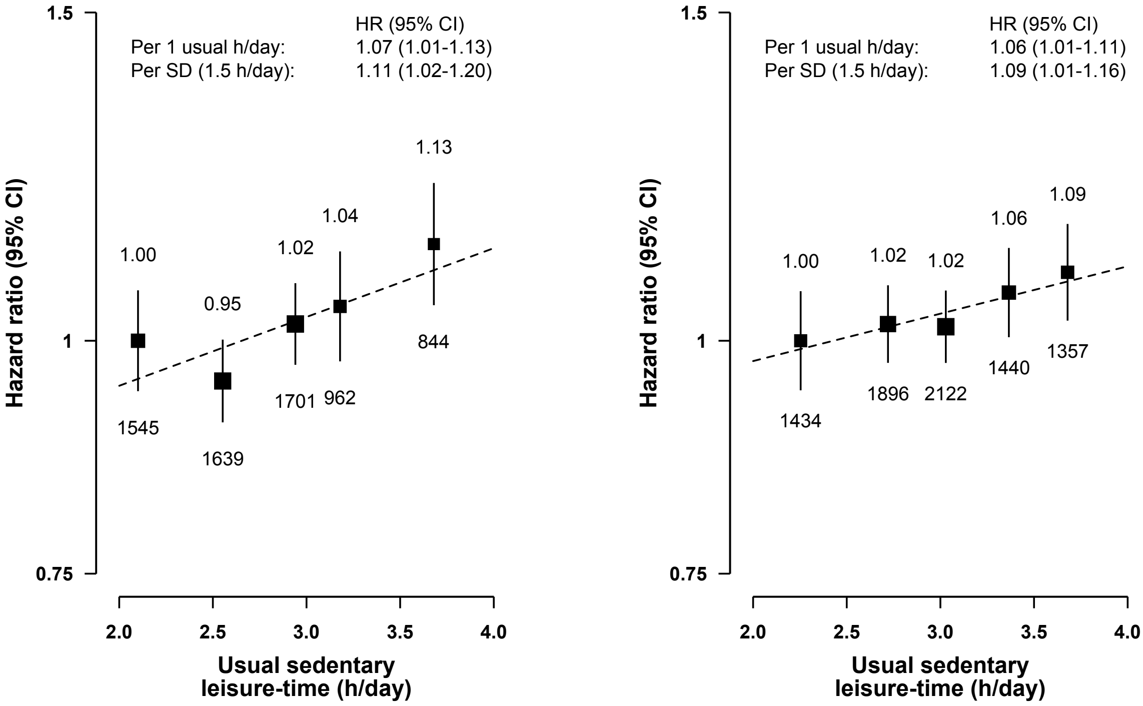

All analyses were stratified by age-at-risk, sex and region and adjusted for household income, education, smoking, alcohol, fresh fruit consumption, self-rated health, family history of diabetes, BMI status (<25, 25-29, 30+ kg/m²) and sedentary leisure-time or total physical activity as appropriate. Results per 1 SD increase are for usual total physical activity and sedentary leisure-time.

Bennett DA, *et al. BMJ Open Diab Res Care* 2019;0:e000835. doi: 10.1136/bmjdrc-2019-000835

eFigure 9: Joint associations of total physical activity and BMI with risk of diabetes

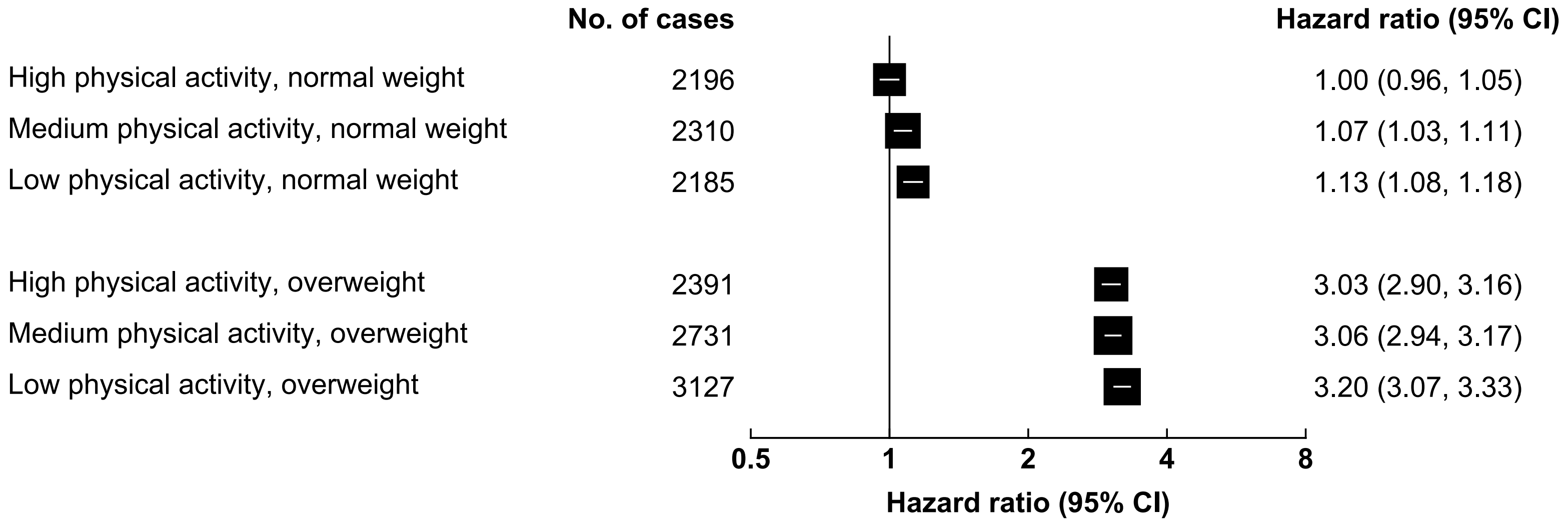

Overweight: BMI ≥ 25 kg/m²   Normal weight: BMI < 25 kg/m²
High physical activity: ≥ 26.4 MET-h/day   Medium physical activity: 13.2 - 26.4 MET-h/day   Low physical activity: < 13.2 MET-h/day

All analyses were stratified by age-at-risk, sex and region and adjusted for household income, education, smoking, alcohol, fresh fruit consumption, self-rated health, family history of diabetes and sedentary leisure-time.

15

eFigure 10: Joint associations of sedentary leisure-time and BMI with risk of diabetes

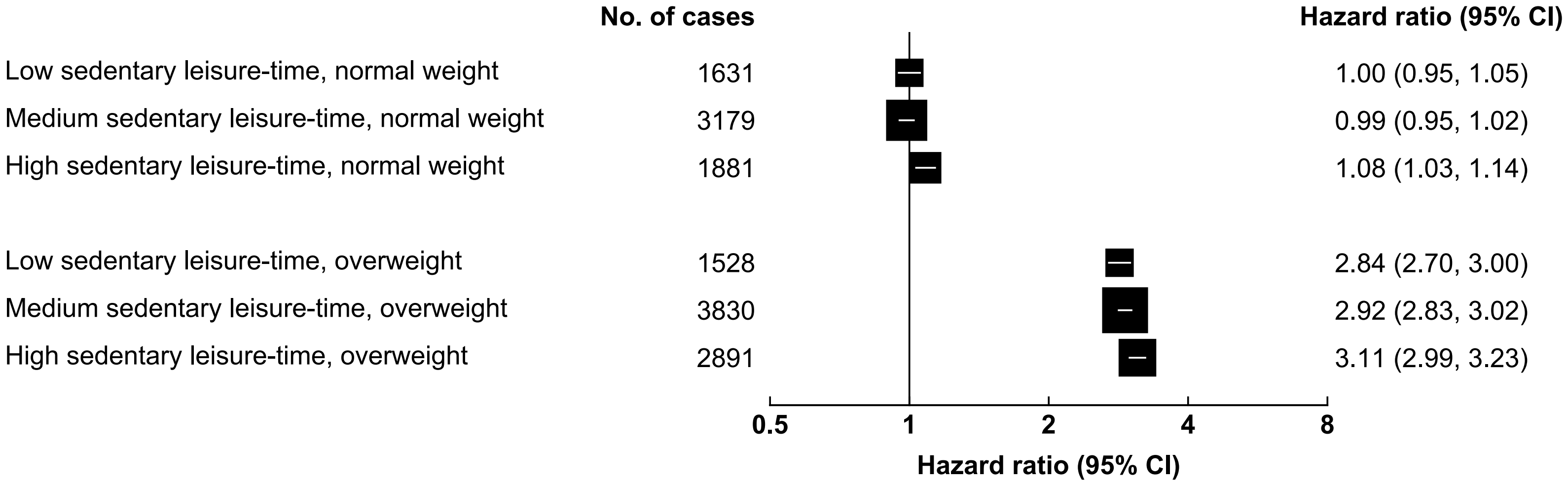

Overweight: BMI ≥ 25 kg/m² **Normal weight: BMI < 25 kg/m²**
High sedentary leisure-time: > 3 h/day **Medium sedentary leisure-time: 2 - 3 h/day** **Low sedentary leisure-time: < 2 h/day**

All analyses were stratified by age-at-risk, sex and region and adjusted for household income, education, smoking, alcohol, fresh fruit consumption, self-rated health, family history of diabetes and total physical activity.

eFigure 11: Adjusted HRs for diabetes per 4 MET-h/day increase in usual physical activity and per 1 h/day increase in usual sedentary leisure-time by subgroup

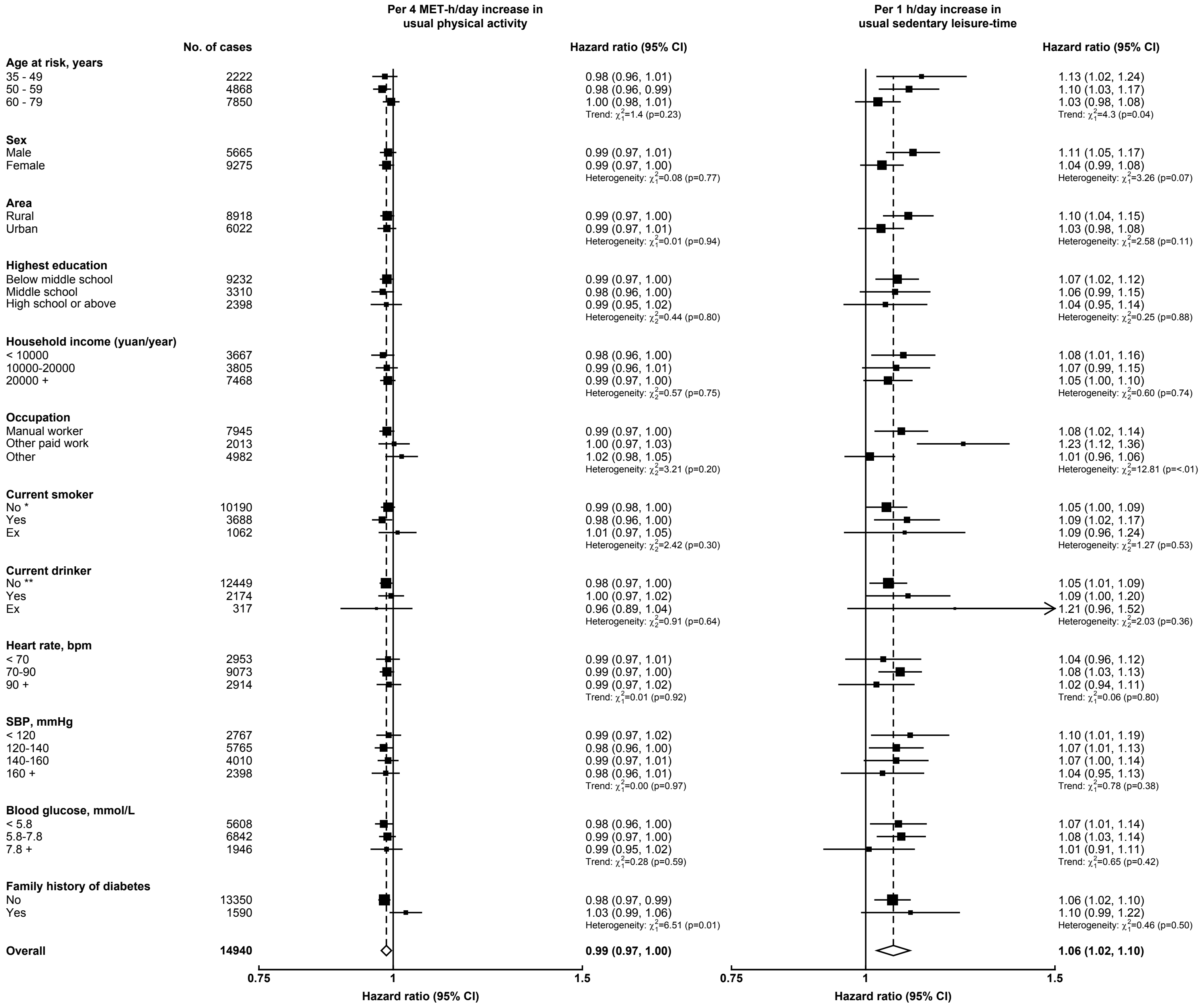

All analyses were stratified by age-at-risk, sex and region and adjusted for household income, education, smoking, alcohol, fresh fruit consumption, self-rated health, family history of diabetes and BMI status (<25, 25-29, 30+ kg/m²).
Adjusted for sedentary leisure-time or total physical activity where appropriate.
* never or occasional, **never regular or occasional or monthly or reduced intake

eFigure 12: Adjusted HRs for diabetes per 4 MET-h/day increase in usual physical activity and per 1 h/day increase in usual sedentary leisure-time by region

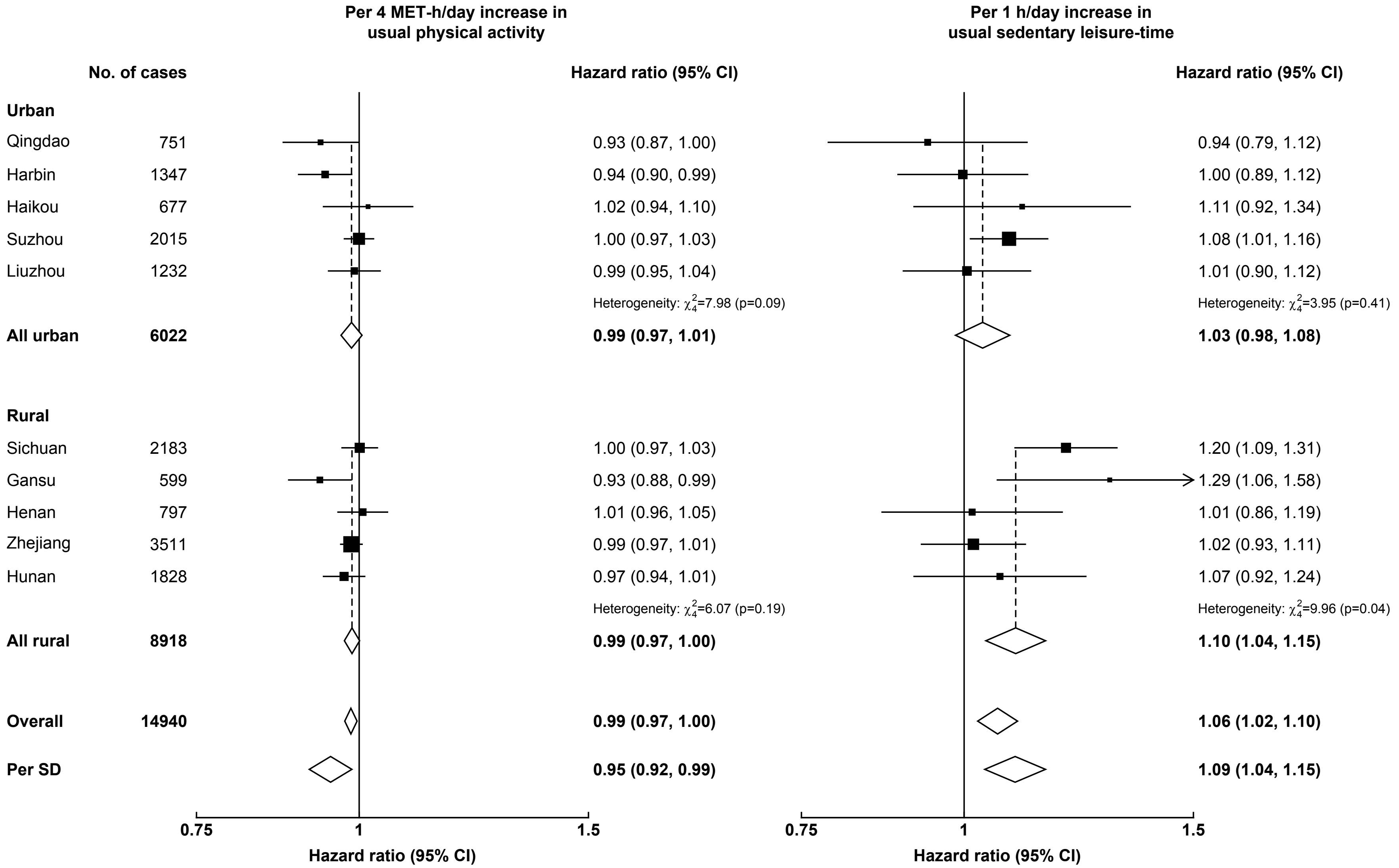

All analyses were stratified by age-at-risk, sex and region and adjusted for household income, education, smoking, alcohol, fresh fruit consumption, self-rated health, family history of diabetes, BMI status (<25, 25-29, 30+ kg/m²) and sedentary leisure-time or total physical activity as appropriate.

eFigure 13: Adjusted HRs for diabetes with total physical activity and sedentary leisure-time after progressive adjustments

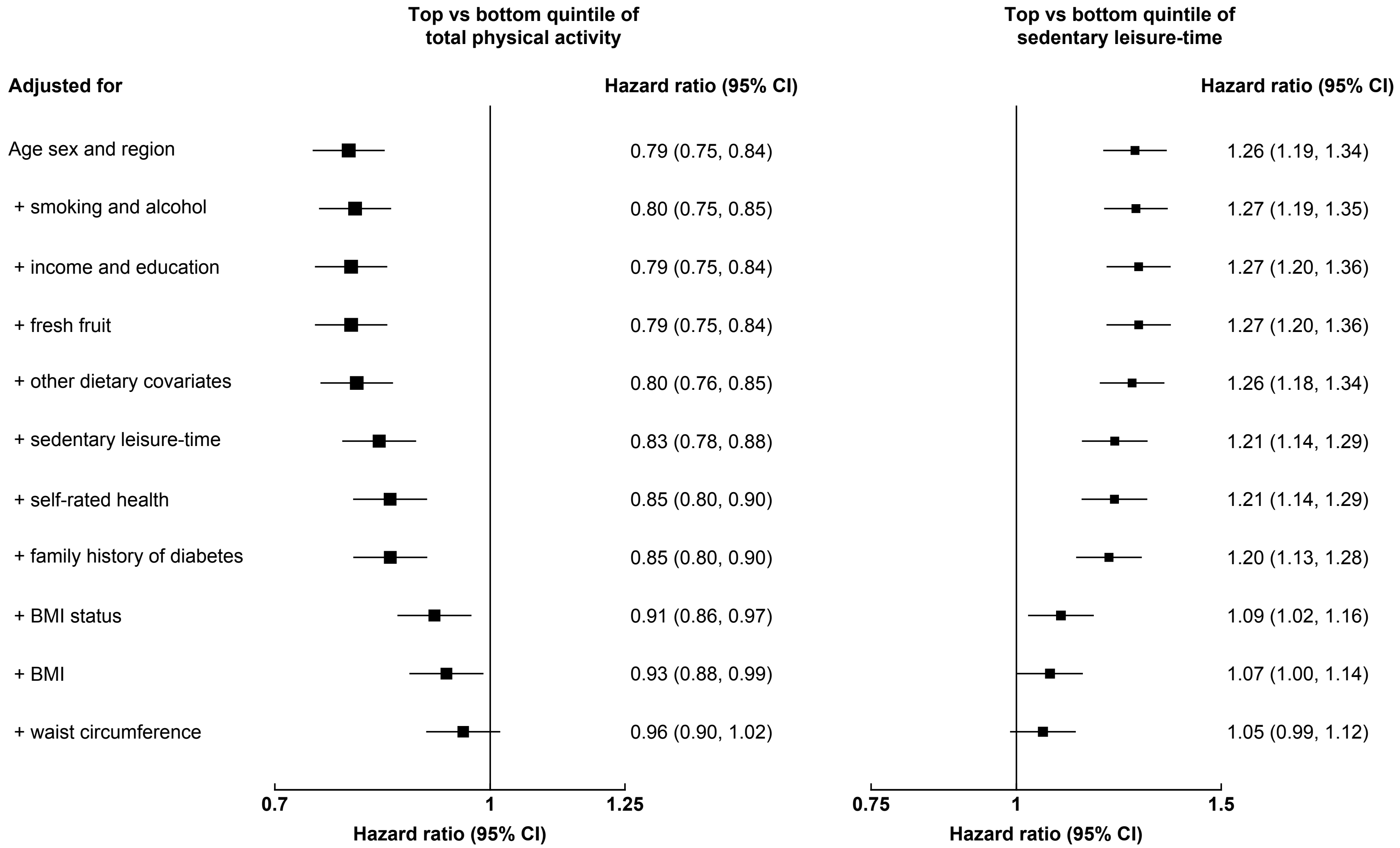

These 95% confidence intervals were not computed using group-specific variances and can be compared to a hazard ratio equal to one for the bottom quintile group.
Other dietary covariates: Consumption of meat, dairy, soybean products, rice, wheat, other staple foods (e.g. corn, millet), poultry, fish, eggs, fresh vegetables, and preserved vegetables.
BMI status: <25, 25-29, 30+ kg/m².
